# LitR and its quorum-sensing regulators modulate biofilm formation by *Vibrio fischeri*

**DOI:** 10.1128/jb.00476-24

**Published:** 2025-01-29

**Authors:** Brittany L. Fung, Karen L. Visick

**Affiliations:** 1 Department of Microbiology and Immunology, Stritch School of Medicine Loyola University Chicago 12248 , Chicago, Illinois, USA; Geisel School of Medicine at Dartmouth, Hanover, New Hampshire, USA

**Keywords:** quorum sensing, *Vibrio fischeri*, biofilms, gene expression

## Abstract

Quorum sensing controls numerous processes ranging from the production of virulence factors to biofilm formation. Biofilms, communities of bacteria that are attached to one another and/or a surface, are common in nature, and when they form, they can produce a quorum of bacteria. One model system to study biofilms is the bacterium *Vibrio fischeri*, which forms a biofilm that promotes the colonization of its symbiotic host. Many factors promote *V. fischeri* biofilm formation *in vitro*, including the symbiosis polysaccharide (SYP) and cellulose, but the role of quorum sensing is currently understudied. Recently, a quorum-sensing-dependent transcription factor, LitR, was shown to negatively influence *V. fischeri* biofilm formation in the context of a biofilm-overproducing strain. To better understand the importance of LitR, we identified conditions in which the impact of LitR on biofilm formation could be observed in an otherwise wild-type strain and then investigated its role and the roles of upstream quorum regulators in biofilm phenotypes. In static conditions, LitR and its upstream quorum regulators, including autoinducer synthases LuxS and AinS, contributed to control over biofilms that were both SYP and cellulose dependent. In shaking liquid conditions, LitR and AinS contributed to control over biofilms that were primarily cellulose dependent. LitR modestly inhibited cellulose transcription in a manner that depended on the transcription factor VpsR. These findings expand our understanding of LitR and the quorum-sensing pathway in the physiology of *V. fischeri* and illuminate negative control mechanisms that prevent robust biofilm formation by wild-type *V. fischeri* under laboratory conditions.

**IMPORTANCE:**

Quorum sensing is a key regulatory mechanism that controls diverse phenotypes in numerous bacteria, including *Vibrio fischeri*. In many microbes, quorum sensing has been shown to control biofilm formation, yet in *V. fischeri*, the link between quorum sensing and biofilm formation has been understudied. This study fills that knowledge gap by identifying roles for the quorum sensing-controlled transcription factor, LitR, and its upstream quorum-sensing regulators, including the autoinducer synthases AinS and LuxS, in inhibiting biofilm formation under specific conditions. It also determined that LitR inhibits the transcription of genes required for cellulose biosynthesis. This work thus expands our understanding of the complex control over biofilm regulation.

## INTRODUCTION

Bacteria produce molecules, known as autoinducers, that can signal whether there are low or high numbers of similar bacteria in the environment (1). When a quorum has been met, changes in gene regulation can occur, allowing groups of bacteria to coordinately alter their behavior in accordance with the density of the bacterial population. This mechanism, quorum sensing (2), is important for control over numerous bacterial processes, such as bioluminescence, virulence factor production, and motility (3). Furthermore, a connection between quorum sensing and biofilm formation, a community-level protective behavior, was initially uncovered in *Pseudomonas aeruginosa* (4), and quorum sensing is now implicated in multiple aspects of biofilm formation in many species of bacteria (5).

**Fig 1:**
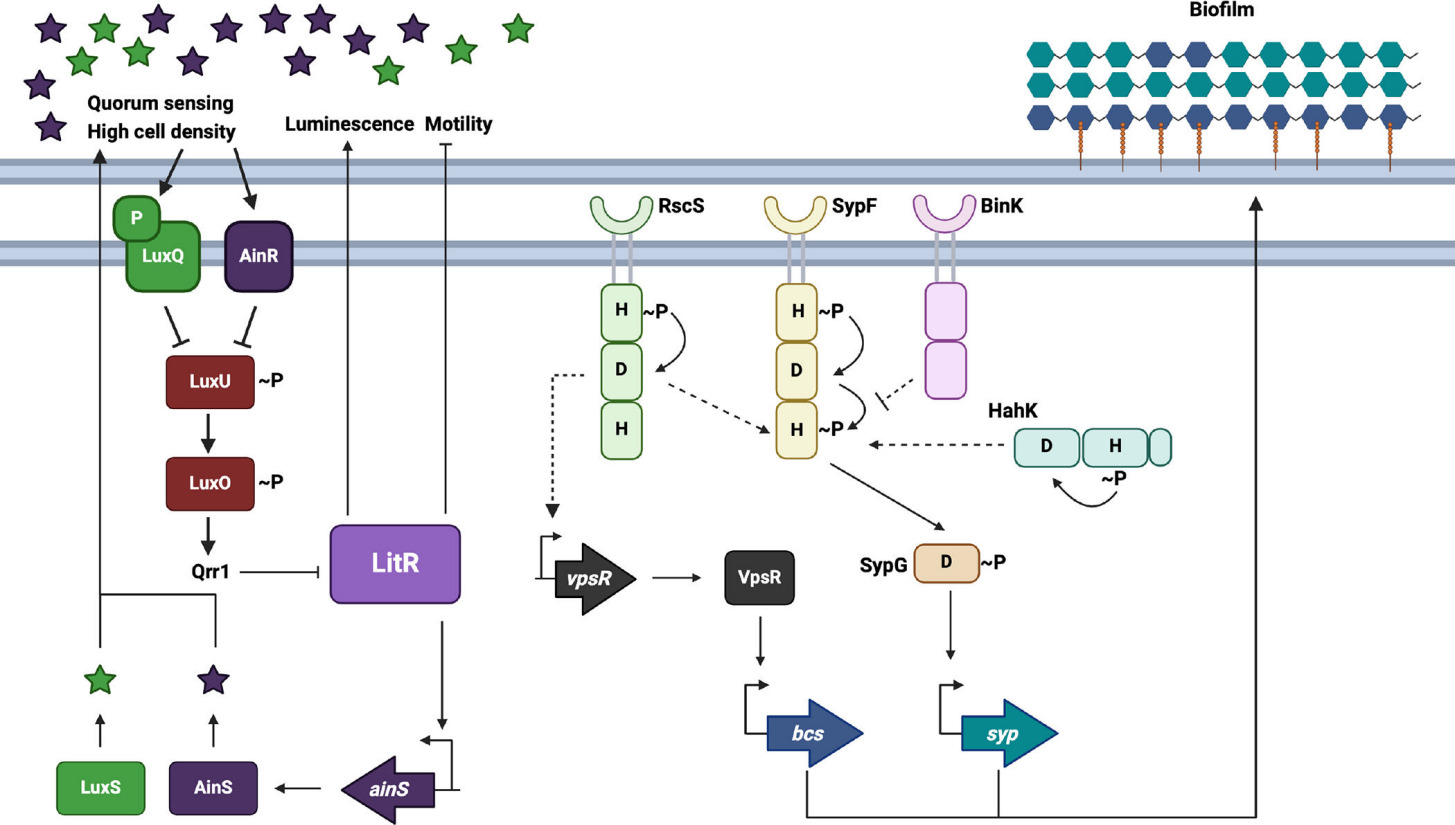
The LitR pathway and relevant biofilm genes. In *V. fischeri*, the transcription factor LitR is produced under high cell density conditions. Under these conditions, autoinducers are produced by the autoinducer synthases LuxS and AinS (light green and dark purple stars) accumulate, which triggers phosphatase activity by their respective histidine kinases, LuxP/Q and AinR (green and dark purple rectangles). The Hpt LuxU is dephosphorylated and removes the phosphoryl group from the response regulator LuxO, preventing LuxO from activating the inhibitory sRNA Qrr1. The relative lack of Qrr1 allows for the translation of *litR* mRNA, and thus, increased LitR production. In a feedback loop, LitR induces *ainS* transcription. LitR enhances bioluminescence and inhibits motility. Two of the known *V. fischeri* biofilm components are SYP (teal hexagons) and cellulose polysaccharide (blue hexagons), along with the surface adhesin LapV (orange line). SYP is controlled at the level of transcription by a suite of regulators. The central histidine kinase, SypF, signals through its Hpt domain to the response regulator SypG, which can induce *syp* transcription. RscS and HahK can also donate phosphoryl groups to SypF’s Hpt domain to induce *syp* transcription, while BinK is predicted to remove phosphoryl groups from SypF’s Hpt domain. Cellulose polysaccharide is transcriptionally controlled by the transcription factor VpsR. Created in BioRender. https://BioRender.com/p78n640.

The marine bacterium *Vibrio fischeri* is a model microbe that is used to study both bioluminescence and biofilm formation, as these traits are important during colonization of its symbiotic host, the Hawaiian bobtail squid, *Euprymna scolopes* (6, 7). In the bioluminescence quorum-sensing pathway, the activities of two autoinducer synthases, AinS and LuxS, indirectly control the phosphorylation status of the response regulator LuxO via two upstream sensor kinase/phosphatases and the phosphotransferase LuxU (8–13). At low cell densities, and thus, low levels of extracellular autoinducer, the phosphorylated form of LuxO (LuxO~P) activates the sRNA Qrr1 to inhibit the production of the transcription factor, LitR (14, 15), a conserved TetR family protein found in other *Vibrios*, including *Vibrio cholerae* (HapR), *Vibrio vulnificus* (SmcR), and *Vibrio harveyi* (LuxR [not the same as *V. fischeri* LuxR]) (16–19). At high cell densities, LuxO is unphosphorylated and no longer active, leading to higher levels of LitR (Fig. 1). LitR then indirectly promotes transcription of the luminescence-producing *lux* operon (15) and via controlling *ainS* (11).

The second well-studied phenotype, biofilm formation, depends on polysaccharides (symbiosis polysaccharide [SYP] and cellulose) that promote cell-cell and cell-surface adherence, respectively (Fig. 1). SYP was identified initially as important for symbiotic biofilm formation and colonization (20–24). It is also required *in vitro* for cohesive biofilm formation (i.e., pellicles and sticky/wrinkled colonies, e.g., references [23, 25–28]). *V. fischeri* controls SYP production using a complex regulatory scheme that involves multiple hybrid sensor histidine kinases (RscS, HahK, and BinK) feeding into controlling the activity of a central sensor histidine kinase, SypF, via phosphorylation/dephosphorylation of its histidine phosphotransferase (Hpt) domain (22, 24, 26, 27, 29–34). SypF controls two downstream response regulators, SypG and SypE, to promote *syp* transcription and SYP production (27, 35). Much of the work that uncovered the SYP regulatory network relied on genetically engineered strains, such as those that overexpressed positive regulators (e.g., RscS, SypF, or SypG) or lacked negative regulators (e.g., BinK or SypE), because they, unlike the wild-type (WT) parent, formed strong pellicles and wrinkled colonies (e.g., references [30, 34, 35]). While useful for establishing pathway components, this type of approach could limit a full understanding of the regulatory network, especially for components that make a more modest contribution.

Similarly, the second polysaccharide, cellulose, has largely been studied in the context of genetically engineered strains. For example, cellulose was shown to be important for both surface adhesion by a hyper-biofilm forming Δ*binK* mutant under shaking liquid conditions and colony bumpiness on agar media (25, 27, 36, 37). Cellulose is produced by proteins encoded by the *bcs* locus. In turn, *bcs* transcription is positively controlled by the transcription factor VpsR and is induced in response to calcium (27, 36, 38).

Several studies have identified connections between quorum sensing and control of biofilm formation in *V. fischeri* (37, 39–41). One study showed that Hpt protein LuxU controls the timing of biofilm production in a SypG-overexpressing, hyper-biofilm forming strain; loss of LuxU delayed the onset of wrinkled colony formation (41). It was speculated that LuxU could donate phosphoryl groups to SypG, thus activating it. Another study revealed that loss of the quorum regulator LitR caused a modest increase in wrinkled colony formation by the Δ*binK* hyper-biofilm former strain (37). This result suggested that LitR inhibits *V. fischeri* biofilm formation. This latter result was consistent with work in *Aliivibrio* (*Vibrio*) *salmonicida*, which discovered a role for the LitR homolog in inhibiting biofilm formation (42). Specifically, the *A. salmonicida* LitR homolog inhibits *syp* transcription, thus controlling biofilm formation. Finally, two other studies (39, 40) demonstrated that *syp* biofilm regulators could control the quorum-sensing pathway by inducing *qrr1* transcription. Together, these studies highlight intriguing connections between these two major regulatory pathways.

Previous work on biofilm formation in *V. fischeri* relied on hyper-biofilm forming strains because the WT parent, ES114, forms biofilms only poorly under standard laboratory conditions (20, 26, 37, 38, 43). Recently, however, conditions that permit ES114 to form *in vitro* biofilms were discovered (25). Specifically, WT produces sticky colonies on a tryptone-based medium (tTBS) supplemented with CaCl_2_ (Ca^2+^) and para-aminobenzoic acid (pABA). Furthermore, some derivatives of WT could form sticky colonies on tTBS plates supplemented with calcium alone (25). These conditions thus avoid the need to use biofilm-overproducing strains, permitting us to probe regulatory connections that may be more subtle and/or otherwise masked in those strain backgrounds.

In this study, we expanded on our previous work by investigating potential mechanisms by which LitR impacts biofilm formation by *V. fischeri*. We first determined which growth conditions resulted in the most robust biofilm phenotypes for an otherwise WT strain in which *litR* is deleted. Subsequently, we used those conditions to probe the regulatory connections that mediate LitR-dependent control over biofilm formation. Finally, we asked if the effect of LitR on biofilm formation is specific to ES114. Our results reveal that quorum sensing, via LitR, controls the formation of biofilms that are primarily cellulose dependent, at least partially at the level of *bcs* transcription. These data thus expand our understanding of the complex impact of LitR on the physiology of *V. fischeri*.

## RESULTS

### LitR inhibits biofilm formation by WT ES114

Past work that identified a role for LitR in inhibiting wrinkled colony formation was performed using a biofilm-overproducing ∆*binK* mutant (37). To gain a better understanding of the role of LitR in controlling biofilm formation, we used a strain that carries a Δ*litR* mutation in an otherwise WT background. We evaluated the Δ*litR* mutant on three different media types (LBS [a yeast extract- and tryptone-containing medium] supplemented with Ca^2+^ [LC], tTBS supplemented with Ca^2+^ [TC], or with Ca^2+^ and pABA [TPC]) and in three different assays of biofilm formation (in static and shaking liquid cultures and on plates).

**Fig 2:**
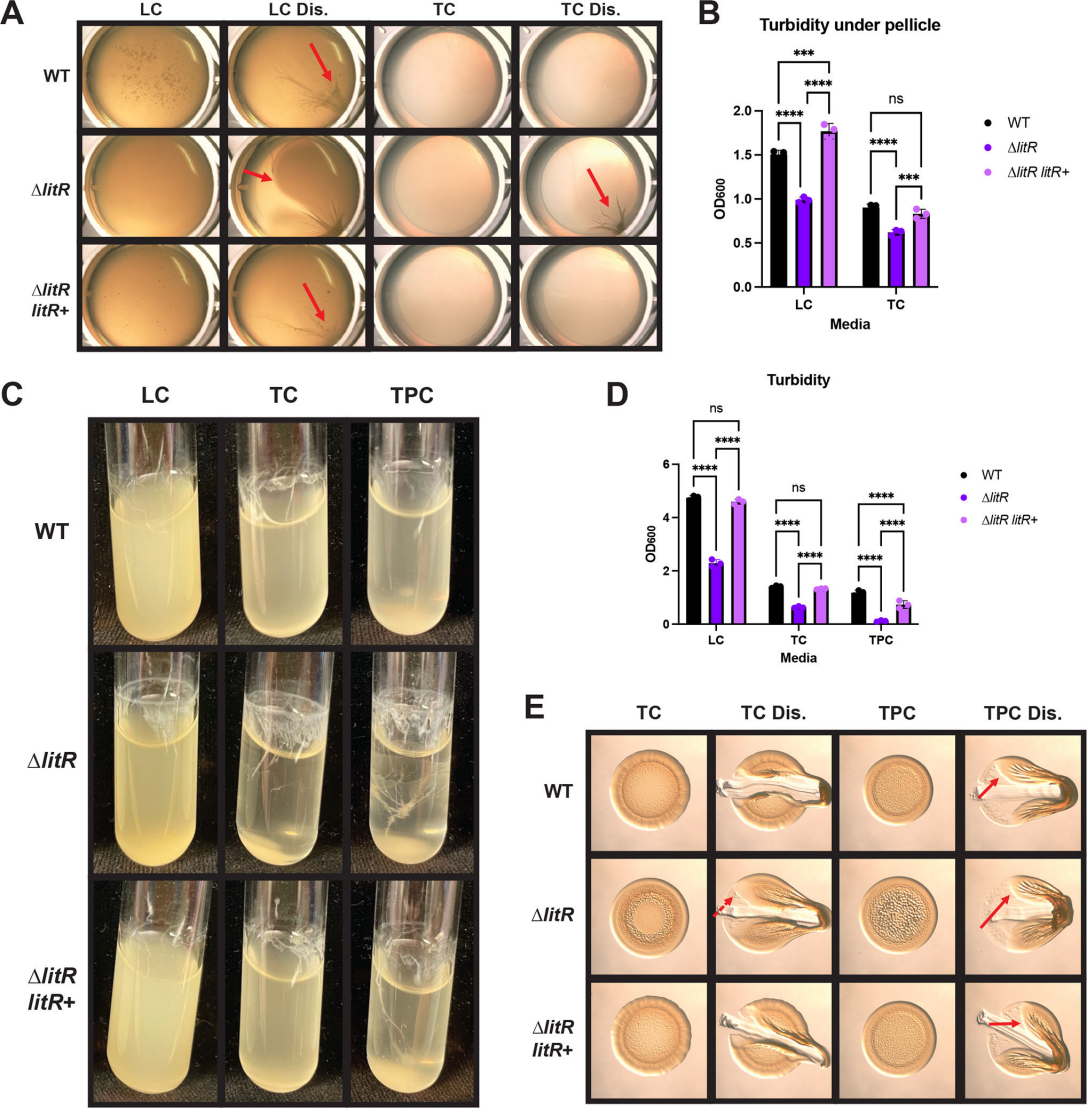
LitR inhibits biofilm formation under static and shaking liquid conditions. WT (ES114), the ∆*litR* mutant (KV10494), and the complemented ∆*litR* mutant (∆*litR litR*+) (BF202) were analyzed in three different growth conditions. (**A**) In static liquid conditions, the strains were assessed after growth in LC and TC for 72 h at 24°C. The pellicles were imaged using the Zeiss Stemi 2000-c microscope at 6.5× magnification and disrupted (Dis.) using a toothpick to assess for stickiness (indicated by the red arrows). (**B**) The turbidity under the pellicle was measured by OD_600_ and plotted. (**C**) Shaking liquid growth was examined in LC, TC, and TPC after incubation for 24 h at 24°C. (**D**) The turbidity of the liquid was measured by OD_600_ and plotted. (**E**) The strains were spotted onto TC and TPC solid agar media and incubated at 24°C for 72 h. The colonies were imaged using the Zeiss Stemi 2000-c microscope at 6.5× magnification and disrupted (Dis.) using a toothpick to assess for stickiness (indicated by the red arrows). The dashed arrows represent minor stickiness. Statistics for panels B and D were performed using a two-way ANOVA, corrected for multiple comparisons using Tukey’s test. ns, not significant. ***P-value < 0.0010 and ****P-value < 0.0001.

We first assessed the role of LitR in biofilm formation using a pellicle assay in which strains were incubated without shaking for 72 h. Biofilm robustness was quantified by measurements of the optical density (OD) of the liquid underneath the pellicle itself; increased biofilm formation inversely correlates with the presence of planktonic bacteria and thus OD and vice versa for decreased biofilm formation. In LC, the ∆*litR* mutant pellicle was stickier and more robust than the WT pellicle. In addition, the edges of the ∆*litR* mutant pellicle were often, although not always, more sharply defined compared to those of the WT, which were typically diffuse (Fig. 2A). The turbidity of the liquid underneath the pellicle was also significantly lower for the ∆*litR* mutant (Fig. 2B); this effect was not due to a growth defect of the ∆*litR* mutant (Fig. S1A). The TC condition also promoted increased pellicle formation for the ∆*litR* mutant relative to the WT, though overall pellicle production was reduced compared to LC (Fig. 2A and B). For both conditions, the increased pellicle formation of the Δ*litR* mutant could be restored to that of the WT by complementation. In TPC, none of the strains tested were able to form pellicles (Fig. S2A, potentially due to the inhibition of flagella-mediated motility that has been identified previously in this growth medium (25). Taken together, it appears that inhibition by LitR contributes to the poor biofilm growth of WT strain ES114 under static liquid growth conditions.

We next grew the cells with shaking, which can result in biofilms in the forms of rings above the liquid interface, adherent strings, and/or clumps at the bottom of the tube (44). As with pellicles, overall biofilm formation can be estimated by measuring the turbidity of the culture, as these phenotypes are inversely correlated. The ∆*litR* mutant exhibited more robust biofilm formation than its parent under all three media conditions tested (Fig. 2C and D). In LC, the ∆*litR* mutant had slightly increased biofilm formation, as visualized by ring and clump formation and decreased turbidity that was not due to a growth defect (Fig. 2C and D; Fig. S1B). In TC, whereas the WT produced only a sparse ring, the ∆*litR* mutant had strings extending from a robust ring, a clump, and correspondingly decreased turbidity. Overall, the phenotypes were more visually striking in TC relative to LC. In both cases, the Δ*litR* phenotypes could be restored to WT levels by complementation (Fig. 2C and D). The ∆*litR* mutant also produced substantial biofilm in TPC, but there was more variability under these conditions, and the increased biofilm of the ∆*litR* mutant was not fully complemented (Fig. 2C and D). These data demonstrate that LitR also inhibits the production of rings, strings, and clumps under conditions of growth with shaking, which is most apparent in TC.

Finally, on TC plates, the ∆*litR* mutant displayed both increased architecture and a very modest increase in stickiness compared to WT (Fig. 2E, left). These results suggest that LitR also contributes to the inhibition of biofilm formation by ES114 on TC plates. On TPC plates, the ∆*litR* mutant exhibited increased colony architecture relative to the WT strain, although the colony stickiness did not differ (Fig. 2E, right). The colony architecture phenotypes were readily complemented on both plate types. Finally, and perhaps not surprisingly, neither WT nor the ∆*litR* mutant exhibited any biofilm phenotype on LC plates; this condition is known not to support WT biofilm formation (25) (Fig. S2B). Together, these data indicate that LitR inhibits biofilm formation, although the extent of this inhibition and our ability to detect biofilm formation are dependent on the growth condition. Therefore, we used TC for shaking and LC for static conditions for the rest of this work because these conditions provided robust phenotypes that could be quantified using OD_600_ measurements.

### Quorum sensing controls LitR’s regulation of biofilm formation

Quorum sensing controls the production of LitR, and ultimately luminescence, via the response regulator LuxO and the sRNA Qrr1 (11, 14, 45, 46) (Fig. 1). It is unclear, however, whether this epistasis holds true for biofilm formation, as prior studies using the ∆*binK* mutant on LC did not reveal a phenotype for Qrr1 (37). Thus, we probed roles for other quorum regulators in *V. fischeri* biofilm formation.

**Fig 3:**
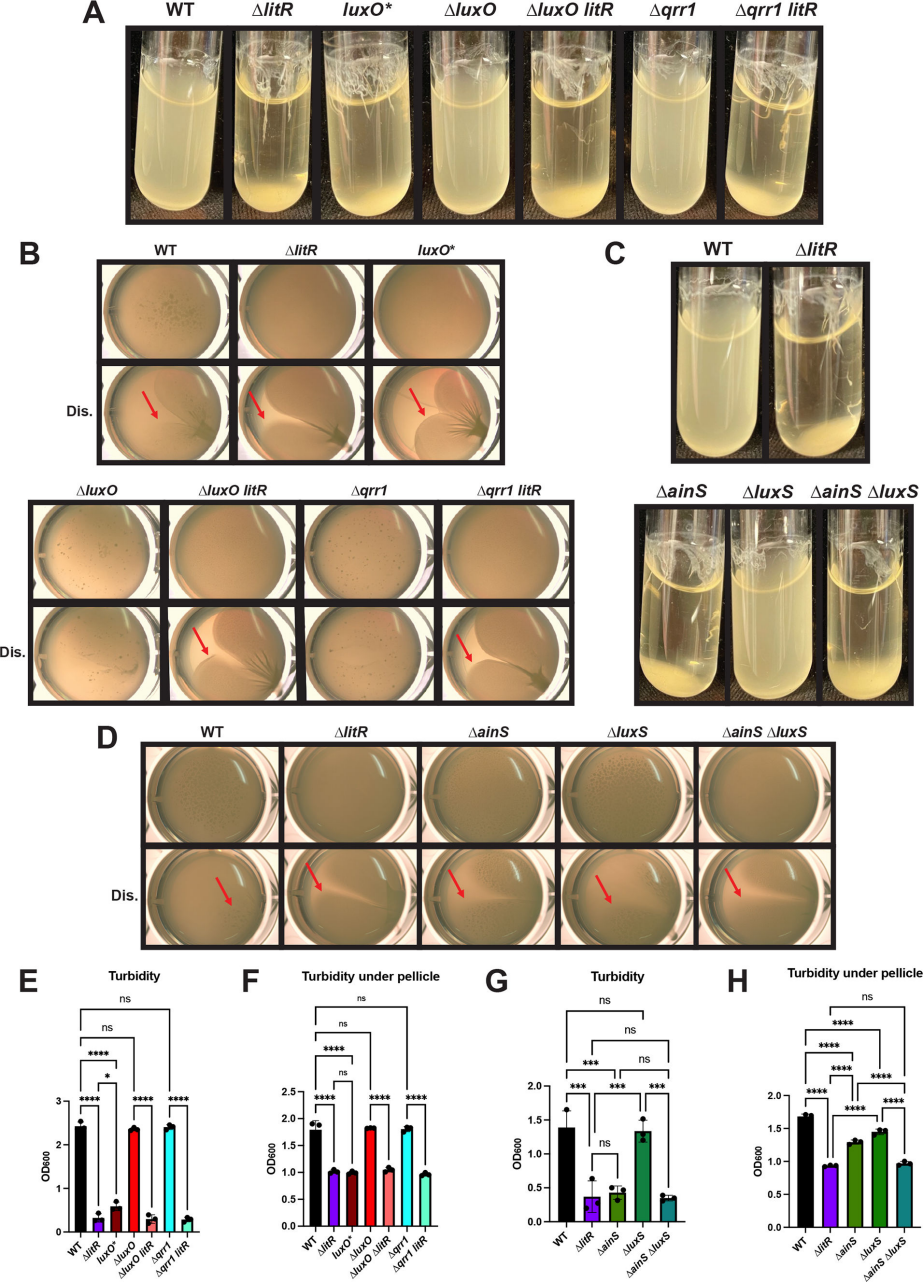
LitR is downstream of the relevant quorum-sensing pathway proteins for biofilm formation. WT (ES114), the ∆*litR* mutant (KV10494), the phosphomimetic LuxO variant (luxO*) (CL59), the ∆*luxO* mutant (KV5467), the ∆*luxO litR* double mutant (KV8791), the ∆*qrr1* mutant (TIM305), and the ∆*qrr1 litR* double mutant (KV8790) were analyzed in (**A**) shaking liquid conditions in TC after 24 h at 24°C and (**B**) static liquid conditions in LC after 72 h at 24°C. WT (ES114), the ∆*litR* mutant (KV10494), the ∆*ainS* mutant (BF430), the ∆*luxS* mutant (CL39), and the ∆*ainS* ∆*luxS* double mutant (CL41) were also assessed in (**C**) shaking liquid conditions in TC after 24 h at 24°C and (**D**) static liquid conditions in LC after 72 h at 24°C. (E and G) Turbidity of the liquid or (F and H) the liquid underneath the pellicle was measured by OD_600_ and plotted. (B and D) Images were taken using the Zeiss Stemi 2000-c microscope at 6.5× magnification. Red arrows indicate stickiness after disruption (Dis.) of the pellicle with a toothpick. Statistics were performed using a one-way ANOVA, corrected for multiple comparisons using Tukey’s test. ns, not significant. *P-value = 0.0172; ***P-value < 0.0004; and ****P-value < 0.0001.

In shaking conditions, a strain of *V. fischeri* encoding a phosphomimetic LuxO variant, a constitutively active form of LuxO, displayed increased biofilm formation, similar to that of the ∆*litR* mutant and consistent with the diminished production of LitR expected under this genetic condition (14) (Fig. 3A and E). In contrast, the ∆*luxO* and ∆*qrr1* mutants did not look phenotypically distinct from WT (Fig. 3A and E). Finally, the ∆*luxO litR* and the ∆*qrr1 litR* double mutants phenocopied the ∆*litR* mutant, suggesting that the ∆*litR* mutation is dominant to the ∆*luxO* and ∆*qrr1* mutations, consistent with the pathway (Fig. 1, 3A, and E).

In pellicle assays, the *lux* pathway regulators functioned as expected from studies of luminescence (12). *V. fischeri* expressing the phosphomimetic LuxO variant exhibited increased pellicle formation, suggesting that Qrr1 inhibits LitR, resulting in enhanced biofilm production (Fig. 3B and F). Correspondingly, the ∆*luxO* and ∆*qrr1* mutants had decreased pellicle formation compared to the WT in static conditions. Finally, the *litR* mutation was epistatic in both double mutant backgrounds (Fig. 3B and F). These results support the conclusion that quorum sensing controls LitR-mediated regulation over biofilm formation.

The activities of two autoinducer synthases, AinS and LuxS, indirectly control the phosphorylation status of LuxO and thus LitR production (Fig. 1); of these, AinS has been shown to be the more impactful with respect to luminescence (9–11). To determine if the same were true for biofilm formation, we evaluated ∆*ainS* and ∆*luxS* mutants. Consistent with the relative impact of these regulators on light production, only the ∆*ainS* mutant exhibited increased biofilm formation under shaking conditions (Fig. 3C and G). While neither the Δ*ainS* mutant nor the Δ*luxS* mutant exhibited strong phenotypes in the pellicle assay, the double ∆*ainS* ∆*luxS* mutant phenocopied the ∆*litR* mutant in both sticky pellicle formation and turbidity underneath the pellicle (Fig. 3D and H). These data indicate that, unlike in shaking liquid conditions, AinS and LuxS both contribute to biofilm control under static conditions, thus supporting the conclusion that these quorum-sensing regulators control LitR under biofilm-forming conditions.

### The ∆*litR* mutant pellicle is dependent on the presence of SYP

**Fig 4:**
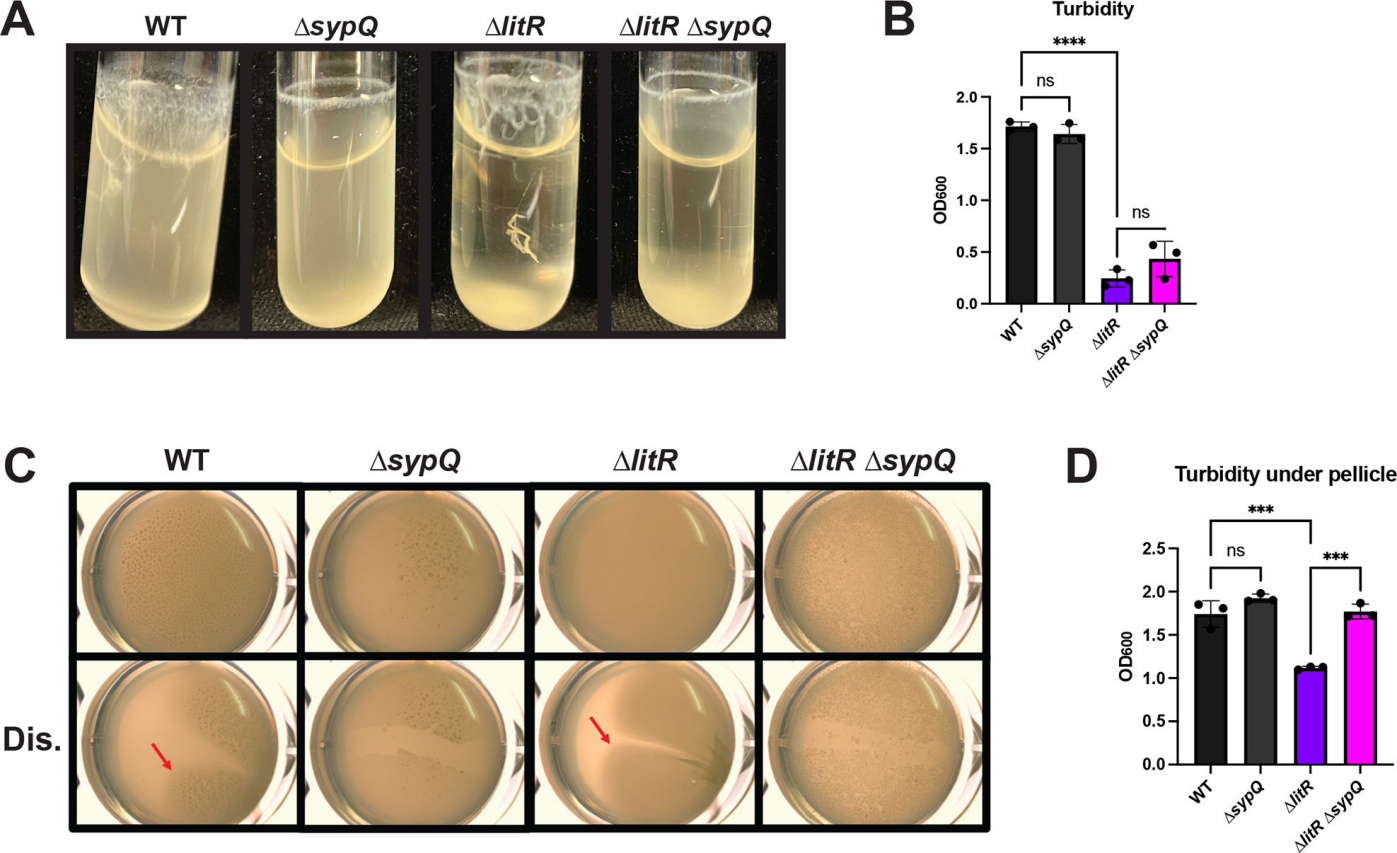
The ∆*litR* mutant is dependent on SYP for its static liquid phenotype. WT (ES114), the ∆*sypQ* mutant (KV8191), the ∆*litR* mutant (KV10494), and the ∆*litR* ∆*sypQ* double mutant (BF13) were assessed in (**A**) shaking liquid conditions in TC after 24 h at 24°C and (**C**) static liquid conditions in LC after 72 h at 24°C. (**B**) Turbidity of the liquid or (**D**) the liquid underneath the pellicle was measured by OD_600_ and plotted. (**C**) The pellicles were imaged using the Zeiss Stemi 2000-c microscope with a magnification of 6.5× with and without disruption (Dis.) to assess for the stickiness of the pellicle (indicated by the red arrow). Statistics were performed using a one-way ANOVA, corrected for multiple comparisons using Tukey’s test. ns, not significant. ***P-value < 0.0002 and ****P-value < 0.0001.

SYP is an important component of the *V. fischeri* biofilm that is responsible for biofilm stickiness or cohesion (25, 47). However, the relative contribution of SYP seems to depend on the growth condition (25). Therefore, to understand whether LitR regulates SYP-dependent biofilm formation, we evaluated the ∆*litR* mutant’s dependence on SYP for its static and shaking liquid phenotypes. To do this, we disrupted *sypQ*, a representative *syp* gene whose loss disrupts SYP production (20). We then tested this strain in shaking and static conditions. In the shaking liquid assay, the ∆*litR* ∆*sypQ* double mutant was phenotypically similar to the ∆*litR* single mutant, except that string formation was abrogated (Fig. 4A and B). We conclude that SYP makes only a modest contribution to biofilm formation under these shaking conditions. In contrast to the results from shaking conditions, pellicles formed by the ∆*litR* mutant under static conditions required SYP (Fig. 4C and D). Although the Δ*litR* Δ*sypQ* double mutant remained competent to form a pellicle, its pellicle was thinner and more fragile than that formed by the ∆*litR* mutant. Thus, pellicles produced by the Δ*litR* mutant depend on SYP production.

### Static biofilm formation is controlled by RscS regardless of the presence of LitR

The *syp* regulatory network has been extensively characterized with the hybrid sensor kinases SypF, RscS, and HahK feeding into the Hpt domain of SypF to activate the response regulator SypG (22, 26, 27, 29, 30, 32, 33). Because SYP was required for the ∆*litR* mutant’s biofilm only in static conditions, we used those conditions to determine the requirement of *syp* regulators for the Δ*litR* mutant pellicle phenotype.

**Fig 5:**
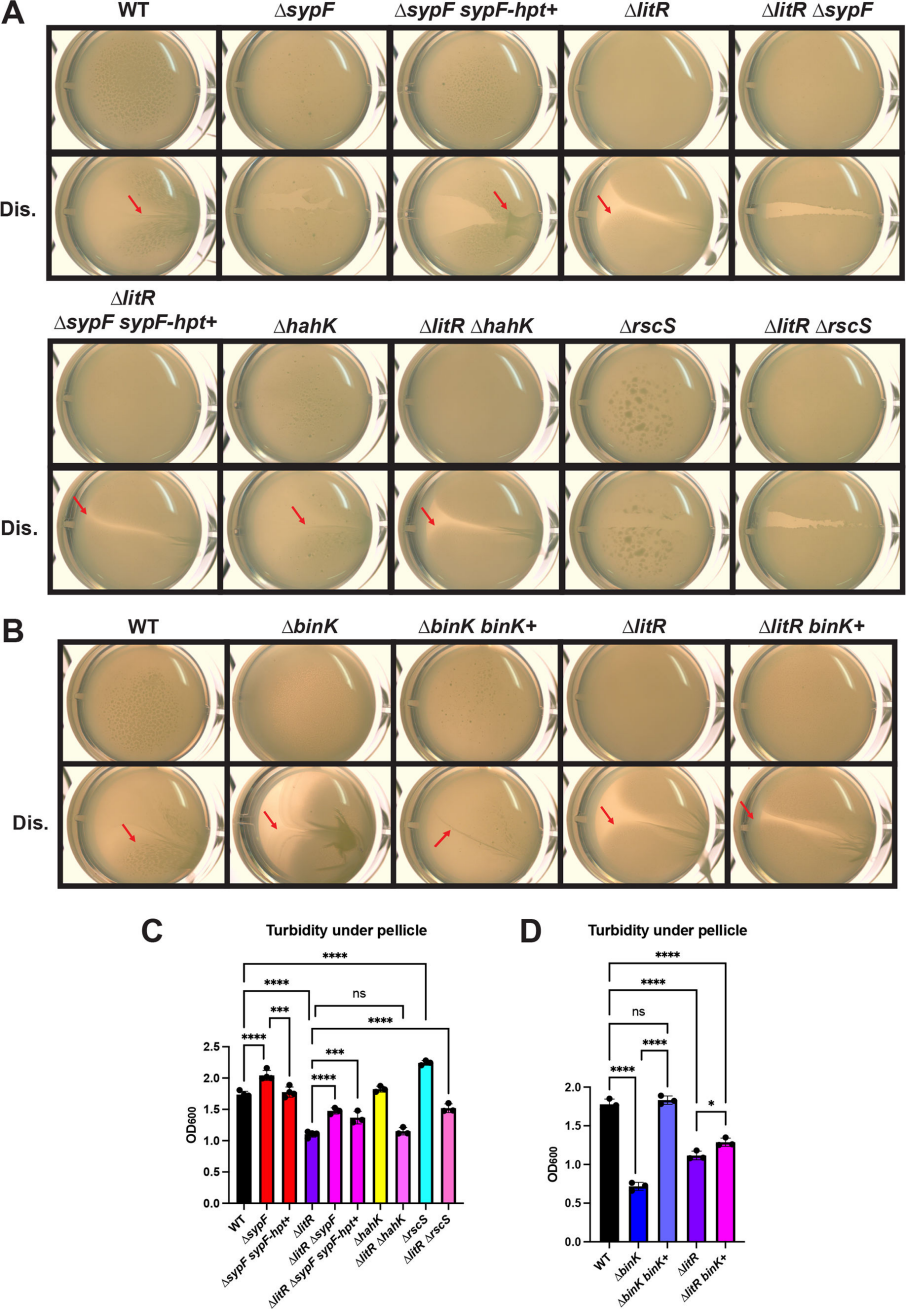
LitR does not control any of the known *syp* regulators for biofilm inhibition in static liquid conditions. (**A**) WT (ES114), the ∆*sypF* mutant (KV8242), the ∆*sypF* mutant expressing just the Hpt domain of SypF (∆*sypF sypF-hpt*+) (KV7226), the ∆*litR* mutant (KV10494), the ∆*litR* ∆*sypF* double mutant (BF247), the ∆*litR* ∆*sypF* double mutant expressing the *sypF-hpt* domain (BF256), the ∆*hahK* mutant (KV10053), the ∆*litR* ∆*hahK* double mutant (BF264), the ∆*rscS* mutant (KV9501), and the ∆*litR* ∆*rscS* mutant (BF263) were examined in static liquid conditions after 72 h of incubation at 24°C in LC. The pellicles were imaged using the Zeiss Stemi 2000-c microscope at 6.5× magnification with and without disruption (Dis.) to assess for the stickiness of the biofilms (indicated by red arrows). (**C**) Turbidity under the pellicle was measured by OD_600_ and plotted. (B and D) Performed as described for panels A and C, using the following strains: WT (ES114), the ∆*binK* mutant (KV7860), the ∆*binK* mutant complemented with *binK* (∆*binK binK*+) (KV9839), the ∆*litR* mutant (KV10494), and the ∆*litR* mutant expressing *binK* (∆*litR binK*+) (BF581). Statistics for panels C and D were performed using a one-way ANOVA, corrected for multiple comparisons using Tukey’s test. ns, not significant. *P-value = 0.0277; ***P-value < 0.0006; and ****P-value < 0.0001.

We first asked how the single ∆*sypF*, ∆*hahK*, and ∆*rscS* mutants performed in the pellicle assay in relation to ES114 as previous work relied on overexpression strains (34, 48). In our LC conditions, the loss of either *sypF* or *rscS* resulted in full disruption of the sticky pellicle and increased turbidity. In contrast, the ∆*hahK* mutant pellicle was not significantly different from that of ES114 (Fig. 5A and C). This suggests that SypF and RscS both contribute to sticky pellicle production by the WT. The critical nature of RscS in pellicle production is noteworthy as few *in vitro* phenotypes have been identified for an *rscS* mutant, and most of those depended on overexpression or mutation of key regulators (26, 29–31).

Because the Hpt domain of SypF integrates multiple signals, including that derived from RscS, we complemented the ∆*sypF* mutant by expressing just its Hpt domain and found that this strain could form a sticky pellicle (Fig. 5A). In addition, the turbidity of the liquid underneath the pellicle returned to WT levels (Fig. 5C). Together, these data suggest that ES114’s sticky pellicle phenotype produced under these conditions is likely due to activation by RscS of SypF’s Hpt domain.

To determine if pellicle formation by the Δ*litR* mutant requires any of these *syp* regulators, we first assessed a ∆*litR* ∆*sypF* double mutant. We found that this strain lost its sticky phenotype, which could be restored by the expression of the SypF Hpt domain alone, suggesting that neither the sensory domain of SypF nor a subset of its signaling domains is required for the ∆*litR* mutant phenotype (Fig. 5A and C). In contrast to the Δ*sypF* result, the ∆*litR* ∆*hahK* double mutant exhibited no loss in biofilm formation compared to the ∆*litR* mutant, suggesting that HahK is not a major factor under these conditions (Fig. 5A and C). Finally, the deletion of *rscS* abrogated the ∆*litR* mutant’s sticky phenotype (Fig. 5A and C), which suggests that robust pellicle production by the Δ*litR* mutant depends on RscS.

Finally, within the *syp* regulatory network is the negative regulator BinK, which is predicted to remove phosphoryl groups from SypF’s Hpt domain to inhibit biofilm formation (24, 31). Indeed, the Δ*binK* mutant formed robust pellicles that could be restored to that of the wild-type parent by complementation with *binK* under the dual control of a non-native and its native promoter (Fig. 5B and D). We hypothesized that LitR could induce *binK* transcription to inhibit biofilm formation. If so, then the presence of a second copy of *binK* under the control of a non-native promoter should suppress the increased pellicle phenotype of the ∆*litR* mutant. We thus introduced the same complementation cassette into the ∆*litR* mutant, such that the strain now contained a second copy of *binK*, and asked if the strain could still form sticky pellicles. We found that this strain (∆*litR binK*+) could form sticky pellicles, although there was a slight increase in turbidity underneath the pellicle (Fig. 5B and D). We conclude that a second copy of BinK is insufficient to restore WT-like biofilm formation to the Δ*litR* mutant.

### LitR does not substantially control *syp* transcription

Given that LitR is a transcription factor, we hypothesized that it could control SYP production by inhibiting *syp* transcription as it does in *A. salmonicida* (42). To test this possibility, we performed β-galactosidase assays with strains that carry the promoter region of *sypA* fused upstream of the *lacZ* gene and a ∆*sypQ* mutation to prevent sticky biofilm formation from affecting OD_600_ readings. Relative to the control in static growth conditions, but not shaking conditions, the ∆*litR* mutant exhibited significantly increased β-galactosidase activity, indicating increased *sypA* transcription (Fig. S3A). These results correspond to the relative importance of SYP under the two conditions (Fig. 4). However, upon further investigation, we determined that the twofold increased activity under static conditions could be observed only when strains additionally contained the ∆*sypQ* mutation; a ∆*litR* mutant with an intact *syp* locus exhibited an increase of only ~2 units of β-galactosidase activity compared to the WT (Fig. S3B). We conclude that, under the conditions of our biofilm assay (an intact *syp* locus), LitR does not substantially inhibit *sypA* transcription.

### The ∆*litR* mutant biofilm is dependent on the presence of cellulose polysaccharide

**Fig 6:**
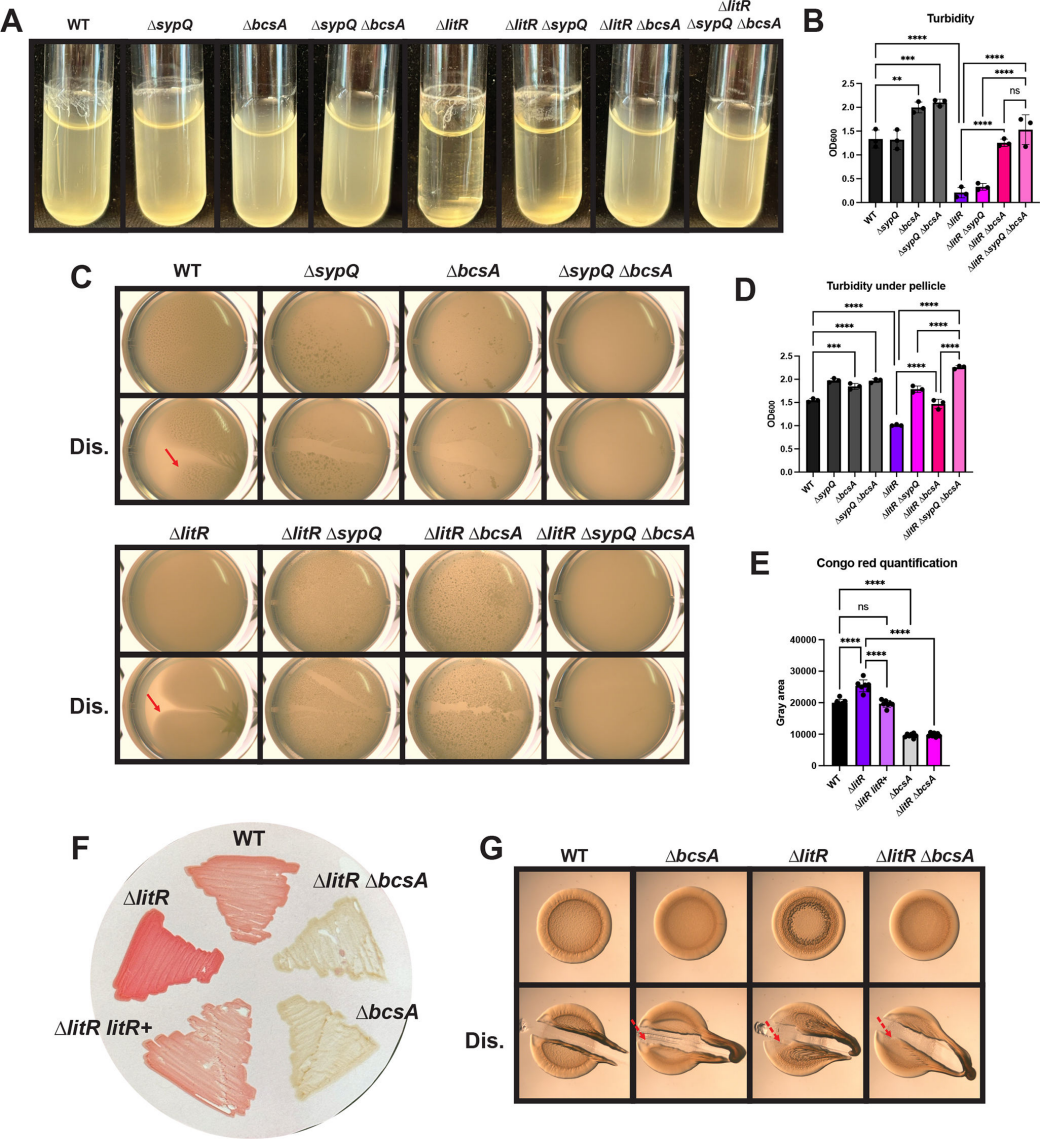
The ∆*litR* mutant is dependent on cellulose for its biofilm phenotype. (A and C) WT (ES114), the ∆*sypQ* mutant (KV8191), the ∆*bcsA* mutant (KV8616), the ∆*sypQ* ∆*bcsA* mutant (KV8753), the ∆*litR* mutant (KV10494), the ∆*litR* ∆*sypQ* mutant (BF13), the ∆*litR* ∆*bcsA* mutant (BF11), and the ∆*litR* ∆*sypQ* ∆*bcsA* mutant (BF211) examined after growth at 24°C in (**A**) shaking liquid conditions at 24 h in TC and (**C**) static liquid conditions at 72 h in LC with and without disruption (Dis.) to assess the stickiness of the pellicle (indicated by red arrows). Pellicles were imaged using the Zeiss Stemi 2000-c microscope at 6.5× magnification. The (**B**) turbidity of the liquid or the (**D**) liquid underneath the pellicle was measured by OD_600_ and plotted. (E and F) Cultures of WT (ES114), the ∆*litR* mutant (KV10494), the ∆*litR litR*+ strain (BF202), the ∆*bcsA* mutant (KV8616), and the ∆*litR* ∆*bcsA* mutant (BF11) were spotted onto tTBS Congo red plates and (E, not shown) left alone or (**F**) spread out over the plate. After incubation for 24 h at 24°C, (**E**) the spots were processed by ImageJ to quantify their color intensity (gray area), and (**F**) the “streaks” were imaged to visualize their redness. (**G**) WT (ES114), the ∆*bcsA* mutant (KV8616), the ∆*litR* mutant (KV10494), and the ∆*litR* ∆*bcsA* mutant (BF11) were spotted onto TC plates and incubated at 24°C for 72 h. The colonies were imaged using the Zeiss Stemi 2000-c microscope at 6.5× magnification with and without disruption (Dis.) using a toothpick to assess stickiness (indicated by red arrows). The dashed arrows represent minor stickiness. Statistics were performed using a one-way ANOVA, corrected for multiple comparisons using the Tukey test. ns, not significant. **P-value = 0.0028; ***P-value < 0.0007; and ****P-value < 0.0001.

Given that LitR impacts biofilm formation but not *syp* transcription, we turned our attention to exploring a role for cellulose, another important polysaccharide, in biofilms produced by the Δ*litR* mutant. Because the role of cellulose in biofilm formation was previously examined only on solid agar plates or in the context of hyper-biofilm-forming mutants (25, 27, 34, 38), we first evaluated its importance in ES114. We found that, relative to WT, the ∆*bcsA* mutant, which is defective for cellulose production, exhibited a reduction of string and ring biofilms in shaking conditions as previously seen in the context of the hyper-biofilm-forming ∆*binK* mutant (34) (Fig. 6A). We also found that the ∆*bcsA* mutant produced less robust pellicles during static growth (Fig. 6C). These results correlated with increased turbidity by OD_600_ of the shaking liquid culture and of the liquid underneath the pellicle, respectively (Fig. 6B and D), indicating that cellulose contributes to the biofilms in these two conditions.

Next, we evaluated the ∆*litR* ∆*bcsA* double mutant in the same conditions. In shaking conditions, the culture of the double ∆*litR* ∆*bcsA* mutant was more turbid than that of its parent, and much of the ring was lost (Fig. 6A and B), indicating biofilms formed by the ∆*litR* mutant under these conditions are reliant on cellulose production. Furthermore, the triple ∆*litR* ∆*bcsA* Δ*sypQ* mutant phenocopied the ∆*litR* ∆*bcsA* double mutant (Fig. 6A and B), which suggests that cellulose is the predominant polysaccharide needed for biofilms produced by the Δ*litR* mutant in shaking liquid conditions. In the pellicle assay, the ∆*litR* Δ*bcsA* mutant exhibited a substantial decrease in biofilm formation relative to the single Δ*litR* mutant. The liquid underneath the pellicle had an increase in turbidity, and the pellicle itself was more fragile (Fig. 6C and D). Because SYP also contributes to the ∆*litR* mutant pellicle, we assessed a triple ∆*litR* ∆*bcsA* ∆*sypQ* mutant and found that it was unable to form a pellicle at all, indicating that the Δ*litR* pellicle depends heavily on both SYP and cellulose polysaccharides (Fig. 6C and D).

We also assessed the contribution of cellulose to the ∆*litR* mutant biofilm by analyzing the strains on plates that contain Congo red, a dye that binds cellulose (as well as amyloid proteins) (49). Previously, the ∆*litR* mutant was assessed on LBS Congo red plates where there was no significant difference compared to the WT (37), correlating with the similar appearance of colonies formed by the two strains on LC (Fig. S2B). In contrast, under tTBS conditions, Δ*litR* mutant colonies exhibited a substantially redder hue, and ImageJ quantification revealed a significant intensity difference relative to the WT (Fig. 6E and F). This phenotype was dependent on the presence of *bcsA*, confirming that the observed red color was due to cellulose (Fig. 6E and F). These data suggest that LitR may inhibit cellulose production under tTBS conditions, correlating with the increased colony architecture observed under similar conditions (Fig. 2E). We thus asked if cellulose was important for the ∆*litR* mutant biofilm on plates. Indeed, the ∆*litR* ∆*bcsA* mutant lost the bumpy architecture of the ∆*litR* mutant, furthering the hypothesis that LitR inhibits cellulose production under tTBS media conditions (Fig. 6G).

### LitR inhibits *bcsQ* transcription

**Fig 7:**
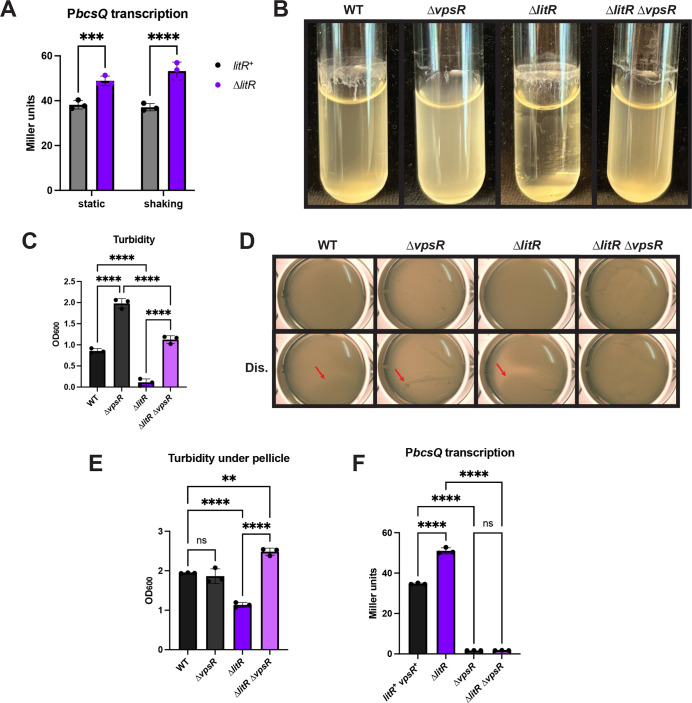
The ∆*litR* mutant is dependent on *vpsR* for its cellulose phenotypes. (**A**) *litR*^+^ (BF255) and ∆*litR* mutant (BF260) strains, both carrying the ∆*sypQ* mutation and the P*bcsQ-lacZ* reporter, were grown with or without shaking for 22 h at 24°C or RT, respectively, in LC. Cell extracts were assayed for β-galactosidase activity, and Miller units were calculated as a measurement of P*bcsQ* activity. (B and D) Biofilms produced by WT (ES114), the ∆*vpsR* mutant (KV9341), the ∆*litR* mutant (KV10494), and the ∆*litR* ∆*vpsR* mutant (BF358) were analyzed after incubation at 24°C in (**B**) shaking liquid conditions for 24 h in TC or (**D**) static liquid conditions for 72 h in LC. (**C**) Turbidity of the liquid or (**E**) of the liquid underneath the pellicle was measured by OD_600_ and plotted. (**D**) The pellicles were imaged using the Zeiss Stemi 2000-c microscope at 6.5× magnification with and without disruption (Dis.) by a toothpick to assess the stickiness of the pellicle (indicated by red arrows). (**F**) *litR*^+^ *vpsR*^+^ (BF255), ∆*litR* (BF260), ∆*vpsR* (BF641), and ∆*litR* ∆*vpsR* (BF643) strains, all carrying the ∆*sypQ* mutation and the P*bcsQ-lacZ* reporter, were grown with shaking for 22 h at 24°C in LC. Cell extracts were assessed for β-galactosidase activity, and Miller units were calculated as a measurement of P*bcsQ* activity. Statistics for panel A were performed using a two-way ANOVA, uncorrected for multiple comparisons with Fisher’s LSD test. ***P-value = 0.001 and ****P-value < 0.0001. Statistics for panels C, E, and F were performed using a one-way ANOVA, corrected for multiple comparisons with Tukey’s test. ns, not significant. **P-value = 0.0012 and ****P-value < 0.0001.

We hypothesized that LitR could exert its effect on cellulose production by controlling *bcsQ* transcription. To test this possibility, we evaluated the β-galactosidase activity of strains that carried the *bcsQ* promoter fused to promoterless *lacZ*. Relative to its parent, the ∆*litR* mutant exhibited increased *bcsQ* transcription under both static and shaking liquid conditions (Fig. 7A). These results indicate that LitR inhibits transcription of *bcs* to control biofilm formation in ES114.

*bcs* transcription is activated by the transcription factor VpsR (36). To begin to probe the mechanism by which LitR controls cellulose production, we first asked if the ∆*vpsR* mutant had a phenotype under shaking and static conditions and found that it did; loss of *vpsR* resulted in increased turbidity and a loss in rings and strings in shaking conditions and a visible difference from the WT pellicle (Fig. 7B through E). To determine if VpsR was required for the biofilms formed by the ∆*litR* mutant, we assessed the ∆*vpsR* ∆*litR* double mutant and found that loss of VpsR disrupted biofilms formed under both shaking and static conditions (Fig. 7B through E), indicating that the Δ*litR* mutant is dependent on VpsR for its biofilms. Thus, we asked if the impact of LitR on *bcs* transcription depends on VpsR and found that it did: loss of LitR did not increase *bcs* transcription in the absence of VpsR (Fig. 7F). Thus, VpsR is epistatic to LitR. Finally, we asked if LitR indirectly controlled *bcs* transcription by regulating the transcription of *vpsR*. However, LitR did not exert any impact on *vpsR* transcription, as measured by β-galactosidase activity (Fig. S4). This result suggests that LitR acts at another level, either directly at the *bcs* locus in a manner that depends on VpsR activity or indirectly through any of a variety of mechanisms, such as controlling VpsR activation. Together, these data demonstrate that LitR inhibits *bcsQ* transcription to reduce cellulose production and thus biofilm formation by *V. fischeri*.

### LitR inhibits biofilm formation in *V. fischeri* KB2B1

To expand our study, we asked if LitR inhibits biofilm formation in another WT strain of *V. fischeri*, KB2B1 (50). This strain readily forms sticky biofilms on both LBS and tTBS media within 72 h (28, 29). Surprisingly, when grown with shaking in TC, KB2B1 had no visible biofilm phenotype (Fig. S5). Furthermore, neither disruption of *litR* nor *qrr1* caused a visible change in cellular aggregation under these conditions (Fig. S5A). Although we measured a small decrease in turbidity for the ∆*litR* mutant (Fig. S5B), the lack of visible biofilm structures indicates that the ∆*litR* mutant does not substantially impact biofilm formation under these conditions.

**Fig 8:**
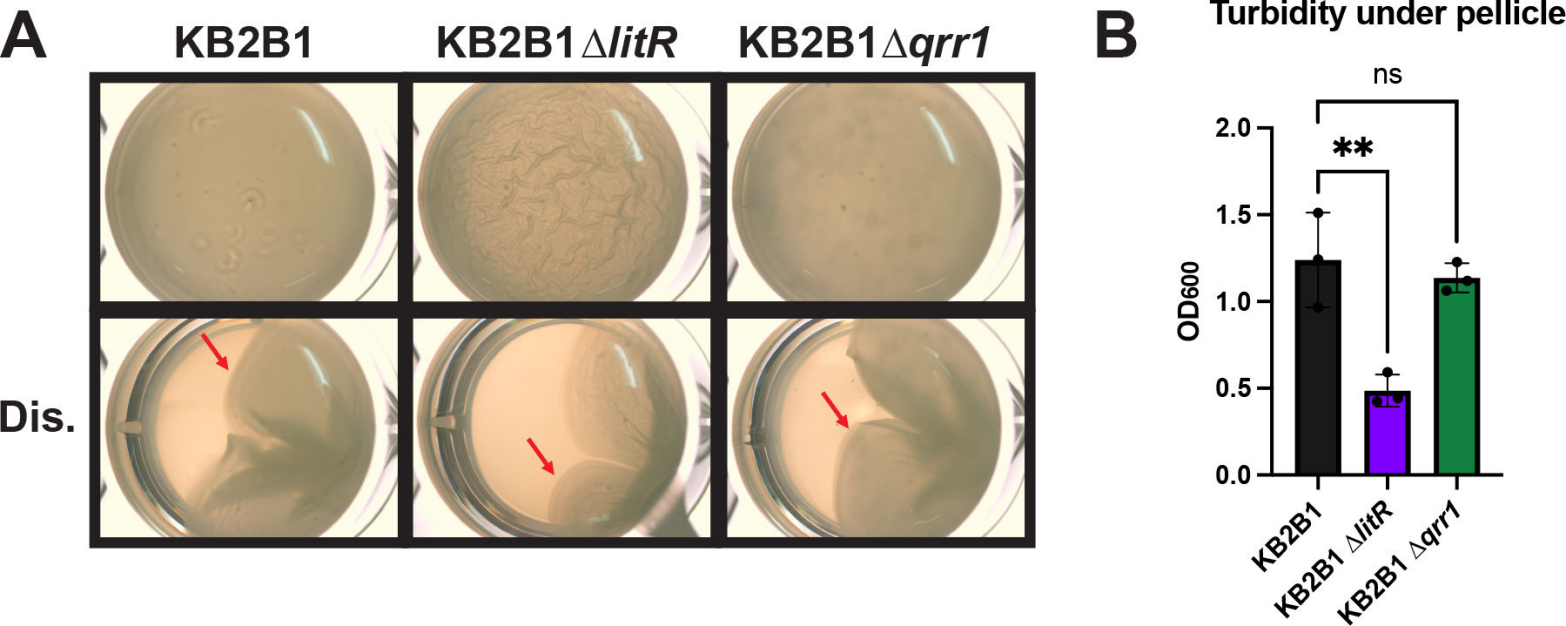
The KB2B1 ∆*litR* mutant has increased static biofilm formation. (**A**) WT strain (KB2B1) and its derivatives, ∆*litR* (KV9023), and ∆*qrr1* (KV9105) were assessed after growth at 24°C in static liquid conditions for 72 h in LC. The pellicles were imaged at 6.5× magnification using the Zeiss Stemi 2000-c microscope with and without disruption (Dis.) using a toothpick to visualize the stickiness of the pellicle (indicated by red arrows). (**B**) Turbidity of the liquid under the pellicle was measured by OD_600_ and plotted. Statistics were performed using a one-way ANOVA, corrected for multiple comparisons using Tukey’s test. ns, not significant. **P-value = 0.0043.

In contrast, in static LBS + Ca liquid conditions, KB2B1 readily formed sticky biofilms (Fig. 8). As with ES114, a null mutation in KB2B1 *litR* resulted in more architecture and reduced turbidity compared to its parent strain (Fig. 8). However, when a *qrr1* mutation, which should lead to increased *litR* translation, was introduced into this strain, there was no change in pellicle formation compared to the control (Fig. 8), as we observed in the shaking conditions for our experiments with ES114 (Fig. 3A and E). Together, our results suggest that LitR also inhibits biofilm formation by KB2B1, at least under static growth conditions.

## DISCUSSION

Quorum sensing is known to control a variety of traits (1, 3). In *V. fischeri*, those phenotypes include luminescence, colonization, acetate metabolism, competence, and motility (11, 12, 15, 45, 46, 51, 52). Here, we expanded our understanding of the role of quorum sensing in *V. fischeri* physiology by probing the ability of the transcription factor LitR and its upstream regulators to influence biofilm formation. Our work revealed two growth conditions under which the negative impact of LitR on biofilm formation was evident, even in the absence of other genetic changes known to increase biofilm formation (e.g., Δ*binK*). Using the static growth condition, we were able to not only visualize a robust increase in biofilm formation by the Δ*litR* mutant but also identify phenotypes for a variety of other mutants, including those defective for other quorum-sensing regulators, as well as for SYP or cellulose regulators whose roles in biofilm formation had been determined largely in the context of “biofilm-up” or other non-WT strain backgrounds.

In its natural habitat, *V. fischeri* must adapt to changes in the environment—whether it is changes in the molecular composition of the ocean or during its transition into the squid (34, 53–56). Indeed, a series of recent publications have documented the impact of nutrient conditions on *V. fischeri* phenotypes, including its ability to form biofilms (25, 34, 57–59). We further expand those findings by showing, among other things, that the ability of Δ*litR* mutant cells to bind to Congo red was dramatically increased relative to its WT parent when grown on tTBS (Fig. 6E and F), but not when grown on LBS (37). However, the importance of specific nutrients appears to be condition dependent. For example, previous work (25) had revealed that yeast extract, when present in agar media, is inhibitory to SYP-dependent biofilm formation by WT *V. fischeri* strain ES114; yet, here we found that one of the conditions that permitted biofilm formation was static growth in the yeast-extract-containing medium LBS (when supplemented with calcium).

It is also evident that *V. fischeri* controls its biofilm formation depending on whether the cells experience static or shaking conditions. For example, aligning with previous work (25, 34), we found that shaking cultures produced biofilms that were largely, although not entirely, dependent on cellulose, while static cultures produced biofilms that depended on both cellulose and SYP. Differential quorum sensing-dependent regulation under shaking vs static growth has been documented before in *P. aeruginosa*, where the sRNA PrrF activates 2-alkyl-4(1*H*)-quinolones to induce type 6 secretion system genes only under static conditions (60). In the context of host colonization, *V. fischeri* experiences both turbulent and static environments: when *V. fischeri* enters the mantle cavity of its squid host, cilia on the surface of the symbiotic organ beat at a higher frequency, creating an environment that is volatile, akin to shaking conditions (61), whereas other areas, called sheltered zones, provide calmer conditions, similar to static growth (62). These varying environments could signal to *V. fischeri* where it is positioned within the squid, allowing the bacteria to switch their genetic regulation and transcribe genes that are important for these different zones. Along those lines, it is of interest that colonization-dominant strain KB2B1 failed to form any biofilm under shaking conditions, yet more robustly formed biofilms under static conditions (Fig. 8; Fig. S5). In the context of its squid host, KB2B1 rapidly forms aggregates that are ~50-fold larger than those of ES114 and colonizes faster than all other WT isolates tested, including ES114 (63, 64). Thus, KB2B1 appears to readily recognize the calm sheltered zones to quickly form enhanced aggregates that position it to successfully initiate colonization. Our results thus suggest that a stronger static biofilm phenotype is more predictive of successful squid colonization than other phenotypes, such as biofilms formed under shaking conditions, correlating with our data that suggest that SYP is an important component of static, but not shaking biofilms (Fig. 4). While we cannot yet distinguish whether the response of *V. fischeri* to these different conditions is due to oxygen sensing or to some other factor, such as surface signaling, this is an important area needing further investigation.

Our work indicated that the Δ*litR* mutant forms biofilms under static conditions that require both the SYP and cellulose polysaccharides. These data align with how LitR behaves in *A*. *salmonicida*, where the ∆*litR* mutant is also dependent on SYP for its static biofilm phenotypes (42, 65). However, while *A. salmonicida* appears to significantly upregulate the *syp* genes, we saw little to no effect on *syp* transcription when disrupting *litR* in an otherwise WT strain background. Of course, both the approach (microarray vs β-galactosidase assay) and the media and growth conditions differed, making it difficult to judge if this is a species-level difference or an artifact of the approach. We did see an effect of *litR* disruption on *syp* transcription when a *sypQ* mutation was also present (Fig. S3). While we confirmed this effect by remaking and retesting our strains, it is unclear what it means. Potentially, the combination of the lack of LitR and of SYP production (resulting from the loss of SypQ) provides a signal for increased *syp* transcription, akin to a stress response. In any case, under the conditions of our biofilm assay (with an intact *syp* locus), LitR did not substantially impact *syp* transcription, suggesting that it exerts its effect on biofilm formation through another pathway(s). Consistent with this possibility, LitR modestly inhibited *bcs* transcription; loss of LitR increased both *bcs* transcription and cellulose-dependent phenotypes.

Mutants defective for quorum-sensing regulators exhibited the biofilm phenotypes expected for their relative positions in the pathway. Similar results were observed in *Vibrio parahaemolyticus*, where LuxO also promotes biofilm formation (66). In *V. fischeri*, by and large, the static assay proved more informative. For example, the ∆*luxO* and ∆*qrr1* mutants exhibited diminished biofilm formation under static conditions, while they phenocopied the WT when grown with shaking. We speculate that the more rapid growth of WT cells under shaking conditions permits them to quickly achieve a sufficiently high cell density such that LuxO is inactive and Qrr1 is no longer produced (14), making the WT indistinguishable from Δ*luxO* or Δ*qrr1* mutants.

The *V. fischeri* quorum-sensing pathway has previously been implicated in biofilm formation, and in turn, biofilm regulators have been shown to provide input into quorum-sensing control. For example, LuxP/Q signals through the Hpt LuxU to not only control luminescence but also activate the response regulator SypG, thus inducing *syp* transcription and SYP production, at least as assayed in a biofilm-up strain (41, 67). In turn, SypG and SypK, a predicted translocase in the inner membrane, directly and indirectly, respectively, upregulate *qrr1* transcription at high cell densities, disrupting LitR synthesis and subsequent control over downstream phenotypes, including luminescence and biofilm formation as well as motility (39, 40). Given that planktonic cells of *V. fischeri* enter into the mantle cavity and then transiently form a small aggregate on the surface of the squid’s symbiotic organ before using flagella-mediated motility to migrate inside, it is likely that multiple inputs signal for a dynamic interaction. Using published and current work and previously suggested models (40, 45) as a foundation, we speculate that the density-sensing sensor kinases such as LuxP/Q in initially low-cell-density bacteria autophosphorylate and donate their phosphoryl groups to LuxU. LuxU (i) inhibits luminescence via LuxO and Qrr1, preventing LitR activity and (ii) provides an initial signal to induce SypG-dependent aggregation (41, 62, 68). As the sensor kinases SypF, RscS, and HahK receive their respective signals within the sheltered zones of the light organ, SypG is further activated, boosting SYP production and reinforcing Qrr1 activation to inhibit LitR and promote biofilm formation. As the cells accumulate to a high cell density in these aggregates, quorum sensing deactivates LuxO and biofilm dispersal signals turn off SypG, disrupting Qrr1 activity and permitting LitR activation, leading to biofilm inhibition, dispersal from the aggregate, and migration into the crypts (39, 40). Consistent with this proposed role of LitR in the transition from aggregation to dispersal, Lupp and Ruby (46) reported that a Δ*litR* mutant exhibits an early (12 h) colonization defect relative to the WT strain. We note that, while LitR paradoxically also functions to inhibit bacterial motility (46, 69), the inhibition is not sufficient to prevent *V. fischeri* from migrating into the crypts. While many aspects of this model remain to be tested, it underscores the growing body of evidence of the interconnectedness of these two major regulatory pathways important for *V. fischeri* colonization.

Overall, by identifying and using different growth conditions to evaluate biofilm formation by *V. fischeri*, this work uncovered LitR’s significant negative effect on biofilm formation. It also highlighted the complexity of control that quorum sensing exerts over biofilm regulation in *V. fischeri*. Finally, it demonstrates that *V. fischeri* is quite sensitive to its growth conditions, underscoring the ready ability of *V. fischeri* to sense and respond to the changing environments it experiences during its lifecycle.

## MATERIALS AND METHODS

### Growth conditions, media, and strain construction

The strains and plasmids used in this study are listed in Tables 1 and 2 and Table S1. *V. fischeri* was maintained as previously described (70). Briefly, for routine culturing, *V. fischeri* was grown in/on LBS (10 g/L tryptone, 5 g/L yeast extract, and 20 g/L NaCl, Tris-buffered to pH 7.5, with or without 15 g/L agar) (70) or tTBS (10 g/L tryptone and 20 g/L NaCl, Tris-buffered to pH 7.5) (25) at 28°C. For biofilm assays, calcium chloride was added to a final concentration of 10 mM to LBS (LC) (34) or tTBS (TC) or calcium chloride (10 mM final), and para-aminobenzoic acid (9.7 mM final) were added to tTBS (TPC) (25). For transformations, *V. fischeri* was grown in Tris-minimal medium (TMM), which contains 100 mM Tris pH 7.5, 300 mM NaCl, 50 mM MgSO_4_, 0.33 mM K_2_HPO_4_, 10 µM ferrous ammonium sulfate, 0.1% NH_4_Cl, 10 mM N-acetylglucosamine, and 10 mM KCl (71). Antibiotic resistance of *V. fischeri* was selected for using the following concentrations: chloramphenicol (Cm, 1 µg/mL), erythromycin (Erm, 2.5 µg/mL), kanamycin, (Kan, 100 µg/mL), spectinomycin (Spec, 200 µg/mL), and trimethoprim (Trim, 10 µg/mL).

**TABLE 1:** Strains used in this study

Strains	Genotype^*a*^^,^^*b*^	Construction^*c*^	Reference
ES114	Wild type	N/A	(72)
KB2B1	Wild type	N/A	(73)
BF11	*litR*::Kan^r^ ∆*bcsA*::FRT-Trim^r^	TT PMF8 with gKV8616	This study
BF13	*litR*::Kan^r^ ∆*sypQ*::FRT-Erm^r^	TT PMF8 with gKV8191	This study
BF202	∆*litR*::FRT IG (Erm^r^)::P*litR-litR*	TT KV10494 with gKV10050	This study
BF211	∆*sypQ*::FRT-Cm^r^ ∆*bcsA*::FRT-Trim^r^ ∆*litR*::FRT-Spec^r^	TT KV8753 with gKV9740	This study
BF237	IG::P*sypA-lacZ* ∆*sypQ*::FRT	Erm^r^ removed from KV9973	This study
BF245	IG::P*sypA-lacZ* ∆*sypQ*::FRT ∆*litR*::FRT-Spec^r^	TT BF237 with gKV9740	This study
BF247	∆*sypF*::FRT ∆*litR*::FRT	Erm^r^, Spec^r^ removed from BF175	This study
BF255	IG::P*bcsQ-lacZ* ∆*sypQ*::FRT	Erm^r^, Cm^r^ removed from BF246	This study
BF256	∆*sypF*::FRT ∆*litR*::FRT attTn*7*::*sypF-hpt-flag*	TT BF247 with gKV7226	This study
BF260	IG::P*bcsQ-lacZ* ∆*sypQ*::FRT ∆*litR*::FRT-Spec^r^	TT BF255 with gKV9740	This study
BF263	∆*rscS*::FRT-Spec^r^ ∆*litR*::FRT	TT KV10494 with gKV9501	This study
BF264	∆*hahK*::FRT-Erm^r^ ∆*litR*::FRT	TT KV10494 with gKV10053	This study
BF315	∆*sypQ*::FRT IG (Erm^r^)::P*vpsR-lacZ*	TT KV9895 with gKV9573	This study
BF330	∆*sypQ*::FRT IG (Erm^r^)::P*vpsR-lacZ* ∆*litR*::FRT-Spec^r^	TT BF315 with gKV9740	This study
BF358	∆*litR*::FRT ∆*vpsR*::FRT	Spec^r^ removed from KV10496	This study
BF430	∆*ainS*::FRT	Erm^r^ removed from KV9367	This study
BF581	∆*litR*::FRT IG (Erm^r^)::P*nrdR*-P*binK-binK*	TT KV10494 with gKV9838	This study
BF624	∆*litR*::FRT IG::P*sypA-lacZ*	Erm^r^, Spec^r^ removed from BF162	This study
BF641	IG::P*bcsQ-lacZ* ∆*sypQ*::FRT ∆*vpsR*::FRT-Trim^r^	TT BF255 with gKV9342	This study
BF643	IG::P*bcsQ-lacZ* ∆*sypQ*::FRT ∆*litR*::FRT-Spec^r^ ∆*vpsR*::FRT-Trim^r^	TT BF260 with gKV9342	This study
CL39	*luxS*::Kan^r^	N/A	(11)
CL41	∆*ainS*::Cm^r^ *luxS*::Kan^r^	N/A	(11)
CL59	*luxO*::D47E^*d*^	N/A	(46)
KV5467	∆*luxO*	N/A	(51)
KV7226	∆*sypF* attTn7::*sypF-hpt-flag*	N/A	(35)
KV7860	∆*binK*	N/A	(34)
KV8191	∆*sypQ*::FRT-Erm^r^	N/A	(74)
KV8242	∆*sypF*::FRT-Erm^r^	TT ES114 with spliced by overlap extension product amplified with primers 1194 and 1160 (ES114), 2089 and 2090 (pKV494), and 2297 and 271 (ES114)	This study
KV8616	∆*bcsA*::FRT-Trim^r^	N/A	(75)
KV8753	∆*sypQ*::FRT-Cm^r^ ∆*bcsA*::FRT-Trim^r^	N/A	(75)
KV8790	∆*qrr1 litR*::Erm^r^	N/A	(51)
KV8791	∆*luxO litR*::Erm^r^	N/A	(51)
KV9023	KB2B1 *litR*::Erm^r^	N/A	(76)
KV9105	KB2B1 ∆*qrr1*::FRT-Erm^r^	N/A	(76)
KV9341	∆*vpsR*::FRT-Spec^r^	N/A	(36)
KV9342	∆*vpsR*::FRT-Trim^r^	TT ES114 with spliced by overlap extension product amplified with primers 2093 & 2094 (ES114) and 2089 & 2090 (pMLC2) and 2095 & 2096 (ES114)	This study
KV9501	∆*rscS*::FRT-Spec^r^	N/A	(29)
KV9573	IG (Erm^r^)::P*vpsR*-*lacZ*	N/A	(36)
KV9839	∆*binK* IG (Erm^r^)::P*nrdR*-P*binK-binK*	TT KV7860 with gKV9838	This study
KV9895	∆*sypQ*::FRT	Erm^r^ removed from KV8191	This study
KV9940	IG::P*sypA-lacZ*	Erm^r^ removed from KV9806	This study
KV10053	∆*hahK*::FRT-Erm^r^	TT ES114 with gKV7952	This study
KV10494	∆*litR*::FRT	Spec^r^ removed from KV9740	This study
TIM305	∆*qrr1*	N/A	(14)

^*a*^ IG, gene inserted at intergenic region between genes *yeiR* and *glmS* along with an FRT scar; IG (Erm), gene inserted between *yeiR* and *glmS* along with FRT-Erm^r^; TT, TfoX-mediated transformation using *tfoX*-overexpressing version of indicated strain; trunc, truncation; RBS, idealized ribosome binding site; FLAG, FLAG-epitope tagged; FRT, flippase recognition target; if not followed by an antibiotic resistance gene, then the antibiotic cassette was flipped out leaving an FRT scar within the chromosome; attTn*7*, site used for insertion of genes using transposon Tn*7*.

^*b*^ All strains are derived from *V. fischeri* isolate ES114 unless otherwise noted.

^*c*^ Strains used for construction are listed in Table S1. Strain construction not applicable (N/A) for already published strains.

^*d*^ Current annotations suggest that the conserved aspartate and corresponding mutation is D55E rather than D47E.

**TABLE 2:** Plasmids used in this study

Name	Description	Reference
pEVS104	Conjugal helper plasmid (Kan^r^)	(77)
pJJC4	*tfoX^+^ +* Cm^r^	(51)
pKV494	pJET + FRT-Erm^r^	(74)
pKV496	*flp*^+^ + Kan^r^	(74)
pKV502	pJET + *yeiR*-FRT-Erm^r^	(74)
pKV503	pJET + *glmS*	(74)
pKV506	pJET + *yeiR*-FRT-Erm^r^-P*nrdR*	(74)
pKV521	pJET + Spec^r^	(74)
pLosTfoX	*tfoX* ^+^	(78)
pMLC2	pJET + Trim^r^	(74)
pLosTfoX-Kan	*pLosTfox* + Kan^r^	(79)

*Escherichia coli* strains were used exclusively for the purpose of promoting genetic manipulation of *V. fischeri*. They were grown in LB (10 g/L tryptone, 5 g/L yeast extract, and 10 g/L NaCl) at 37°C. Antibiotic resistance of *E. coli* was selected for using the following concentrations: Cm (12.5 µg/mL) and Kan (50 µg/mL). The *E. coli* strain π3813 (80), which was used to deliver conjugation helper plasmid pEVS104 (77) and flippase plasmid pKV494 (74), was grown in LB supplemented with 0.3 mM thymidine.

*V. fischeri* genetic manipulation was performed as previously described (71). Briefly, genomic DNA or DNA amplified by polymerase chain reaction spliced by overlap extension (81) was inserted into *V. fischeri* by TfoX-mediated transformation (51). For gene deletions, the endpoints of the deletions are defined by specific primers listed in Tables 1 and 3 and Table S1. Similarly, the endpoints of insertions or promoter-*lacZ* fusions are defined by the primers used. Transformation was accomplished by growing strains that carried a *tfoX* overexpression plasmid in TMM to the mid-exponential phase, then adding DNA, followed by recovery and plating on selective media. Tri-parental conjugations were performed as previously described (71) to introduce *tfoX*-overexpression plasmids or the flippase plasmid pKV496 (74), the latter of which was used to resolve antibiotic resistance cassettes.

**TABLE 3:** Primers used in this study

Primer number	Sequence^a^
271	CTCGGCGCATACTTCTTTAC
1160	taggcggccgcacttagtatgGATGCACTGAATAATTGAGATACC
1194	TTATGTGCGAGGCCTAATGC
1259	GCAATGGTTGAGATCATGTAAA
1487	GGTCGTGGGGAGTTTTATCC
1852	ggcggtaccAGAACCAAGACCTGCTCGTGCTAA
2089	CCATACTTAGTGCGGCCGCCTA
2090	CCATGGCCTTCTAGGCCTATCC
2093	ATCACAGCTCTTGAGCATGG
2094	taggcggccgcactaagtatggTTGAGTACCCATAACACTACCTC
2095	ggataggcctagaaggccatggAGCTATAGCTAATCGAATCCTTATTG
2096	CTGGCAGTAAACCTTTACCTG
2185	CTTGATTTATACAGCGAAGGAG
2196	TCCATACTTAGTGCGGCCGCCTA
2290	AAGAAACCGATACCGTTTACG
2297	ggataggcctagaaggccatggAAACAAGGTTTCTCAAAATAAAAG
2497	taggcggccgcactaagtatggaACGCCAACACTCGTTAAACG
2822	AGGAAACAGCTATGACCATGATTACGGATTCAC
2839	taggcggccgcactaagtatggTCTTGCATTGATCAGTTGTTGAAG
2840	ggataggcctagaaggccatggGAAAAAAACTTCGAAATCAGTGAG
2876	GAAACGCCGAGTTAACGCC
2934	ggataggcctagaaggccatggGGCCATAGTTTGCTCCAG
2935	catggtcatagctgtttccTTCATATCGCTCCTGGGCATTAG
2936	ggataggcctagaaggccatggGTGGCATGACGATCACTC
2937	catggtcatagctgtttccTCATAGTACGGATTCTTTACTTATCG
3014	ggataggcctagaaggccatggTCATTTTGAGCAAAGCGACGG
3017	CAACCATTACTAATCCTTCAG
3018	taggcggccgcactaagtatggCATTATATTTATATCCTTGCCAAC
3019	ggataggcctagaaggccatggTAATTTCAGGATTCATGAAATG
3020	CAATTGCTTACACTGAGCCAG
3083	ggataggcctagaaggccatggAGCTTCTTCCTTATAGTTATGATG
3084	catggtcatagctgttTCCTAGGGAATAATCCTCGTTGTTTC
3310	taggcggccgcactaagtatggATTATTTATAAATACACAACATATTTAAGAAAC
3354	ggataggcctagaaggccatggACTATCTCACTTATTCGTTGAACC

^a^ Lowercase letters represent tail sequences.

### Pellicle assay

*V. fischeri* strains were grown overnight with shaking in LBS or tTBS at 24°C. The resulting OD_600_ was measured and then normalized to 0.02 in 2 mL of LC, TC, or TPC in a 24-well plate. Each strain was inoculated into three separate wells. The plates were incubated at 24°C for 72 h before the wells were imaged on the Zeiss Stemi 2000-c microscope at a magnification of 6.5× before and after disruption using a toothpick, which permitted an assessment of pellicle stickiness, providing an estimate of SYP involvement (47, 75). All images were cropped in the same way to yield comparable results. Following the disruption, the OD_600_ of the liquid underneath the pellicle, avoiding any biofilm, was measured.

### Shaking culture biofilm assay

After overnight growth in LBS or tTBS at 28°C, strains were inoculated in triplicate into 2 mL of fresh LC, TC, or TPC in a 13 × 100 mm test tube at an OD_600_ of 0.05. They were then incubated with shaking at 24°C for 24 h. At this time point, the cultures were imaged using a phone camera, and the OD_600_ of the liquid, avoiding any biofilm, was measured (34). All images were cropped in the same way to yield comparable results.

### Wrinkled colony assay

Strains were grown overnight at 28°C in tTBS or LBS, then subcultured 1:100 into fresh tTBS or LBS for 2 h. The cultures were normalized to an OD_600_ of 0.2, spotted (10 µL) onto 1-day-old TC, TPC, or LC plates, and incubated at 24°C. After 72 h, the strains were imaged using the Zeiss Stemi 2000-c microscope at a magnification of 6.5× and disrupted using a toothpick to assess the stickiness of the colonies (47, 70). All images were cropped in the same way to yield comparable results.

### β-galactosidase assay

After overnight growth in LBS at 24°C, strains were inoculated 1:100 in 20 mL of LC in 125 mL flasks. The cultures were incubated with or without shaking at 24°C or room temperature, respectively, for 22 h. Two milliliters of the cells was spun down and extracted to perform the β-galactosidase assay (Miller assay) as previously described (34, 82). Once the reaction was stopped by the addition of Na_2_CO_3_, the OD_420_ and OD_550_ were both measured to calculate the final Miller units.

### Congo red assay

Strains were grown with shaking overnight in tTBS at 28°C. Twenty microliters of the overnight cultures was spotted on 3-day old Congo red plates (tTBS plates + 40 mg/L Congo red + 15 mg/L Coomassie blue) (83). These spots were left alone or spread over an area and then left to dry before incubation at 24°C. After 24 h, the redness of the spots/streaks was visualized by placing a white paper over the plate and lifting it off with the bacteria (74). The color intensity, measured as the gray area, of each spot was then quantified using ImageJ (36, 84, 85).

### Statistics

All experiments in the main document were performed with at least three biological replicates and representative images are shown. All experiments in the supplement were performed with at least two biological replicates, and representative images are shown. All statistics were performed using GraphPad Prism version 10.1.0. For each strain in all graphs, the mean of three replicates was plotted with the error bars representing the standard deviation.

## SUPPLEMENTAL MATERIAL

Supplemental figures and table jb.00476-24-s0001.pdf Fig. S1 to S5; Table S1.

ASM does not own the copyrights to Supplemental Material that may be linked to, or accessed through, an article. The authors have granted ASM a non-exclusive, world-wide license to publish the Supplemental Material files. Please contact the corresponding author directly for reuse.
