# Supplementary material for: LitR and its quorum-sensing regulators modulate biofilm formation by Vibrio fischeri
Source: J Bacteriol. 2025 Jan 29;207(2):e00476-24. doi: 10.1128/jb.00476-24 (PMC11841056; doi:10.1128/jb.00476-24)
Supplement: Supplemental figures and table — Fig. S1 to S5; Table S1. [file jb.00476-24-s0001.pdf]

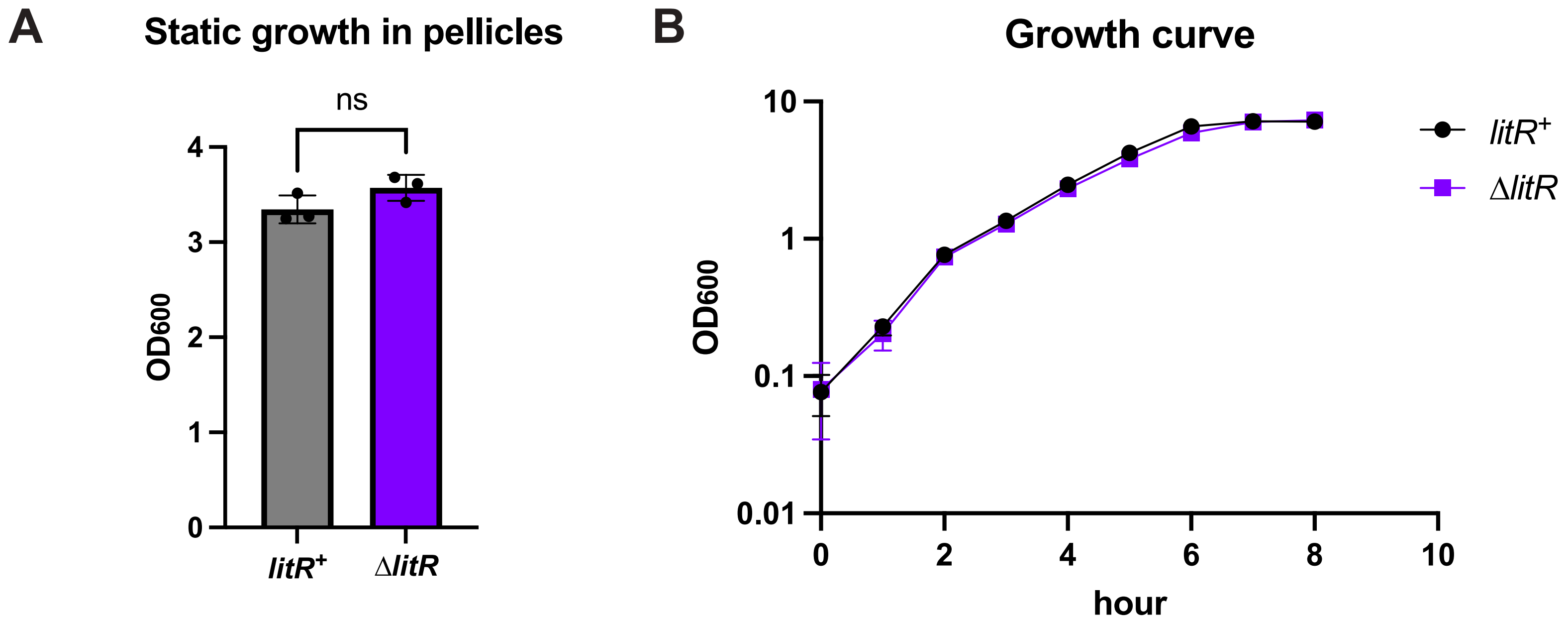

Supplemental Figure 1. The Δ*litR* mutant does not exhibit a growth defect. *litR*$^{+}$ (KV9895) and Δ*litR* mutant (BF13) strains, both of which carried the Δ*sypQ* mutation, were grown at 24°C (A) for 72 h in 24-well plates in LC, at which point the cultures were resuspended and the OD600 was measured or (B) with shaking for 8 h in LC where the OD600 was measured at each hour. Statistics for panel A were performed using an unpaired t test, ns: not significant. Some error bars cannot be seen for panel B because they are shorter than the size of the symbol.

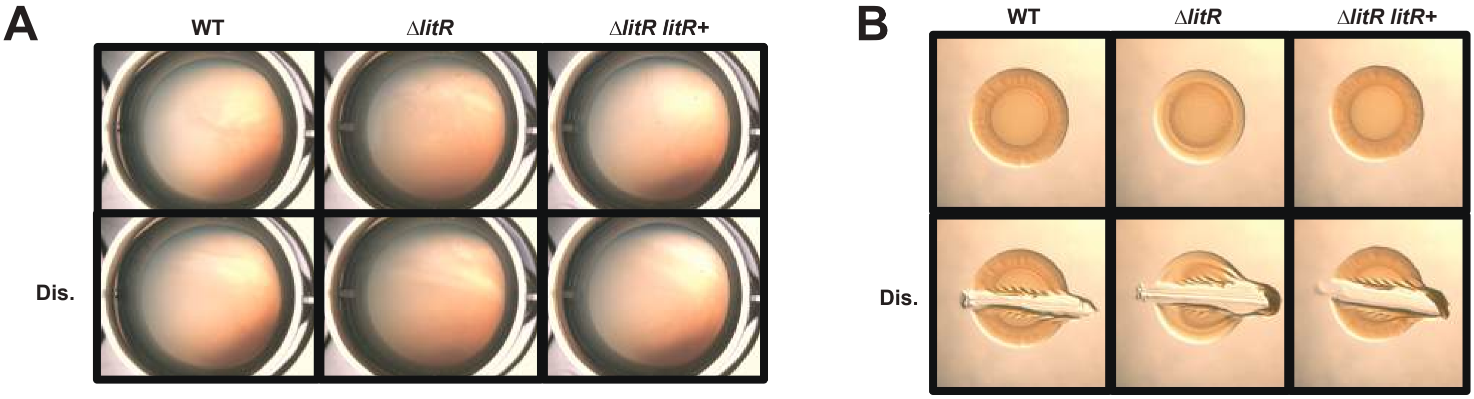

Supplemental Figure 2. The Δ*litR* mutant phenocopies the WT and complemented Δ*litR* mutant strains in static liquid TC and solid agar LC plates. WT (ES114), the Δ*litR* mutant (KV10494), and the complemented Δ*litR* mutant (BF202) were imaged using the Zeiss Stemi 2000-c microscope at 6.5x magnification with and without disruption (Dis.) using a toothpick to assess for stickiness after growth at 24°C for 72 h (A) in static liquid conditions in TC and (B) on solid agar LC plates.

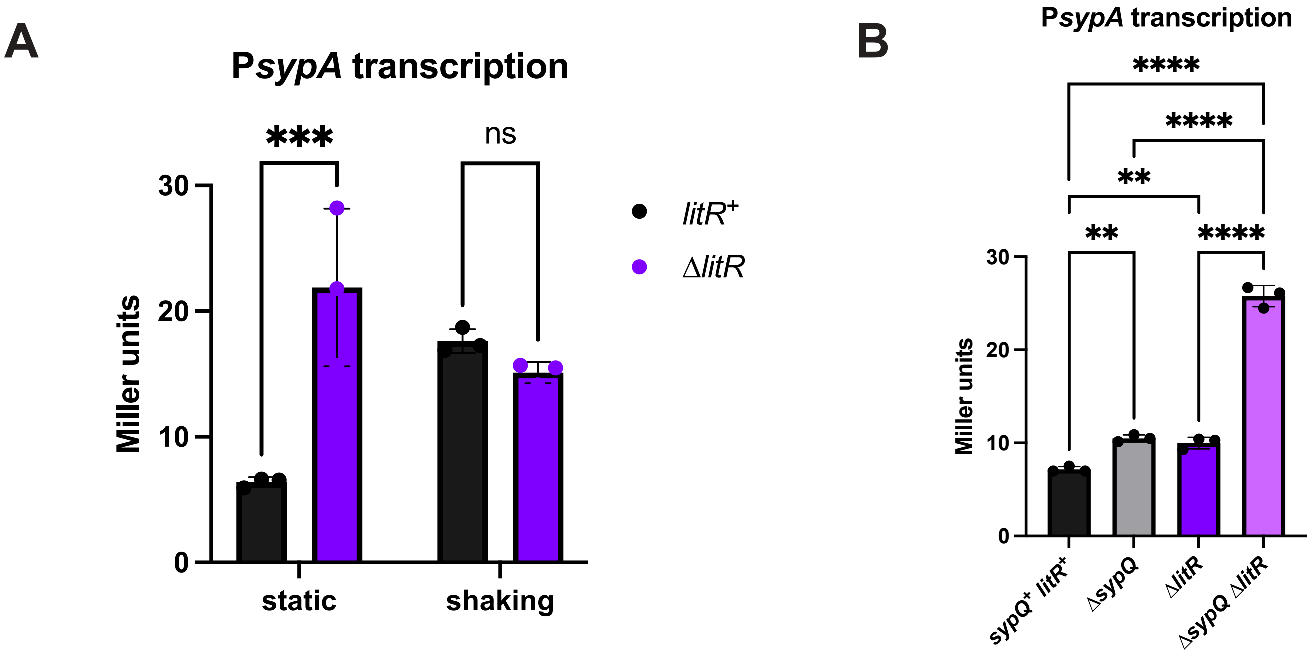

Supplemental Figure 3. The Δ*litR* mutant only affects *sypA* transcription when it also has a Δ*sypQ* mutation. (A) *litR*+ (BF237) and Δ*litR* mutant (BF245) strains, both of which carried the Δ*sypQ* mutation and the P*sypA*-*lacZ* promoter, were grown with and without shaking at 24°C and RT, respectively, for 22 h in LC. A β-galactosidase assay was performed using cell extracts and the Miller units were calculated as a measure of P*sypA* activity. (B) *litR*+ (KV9940), Δ*litR* mutant (BF624), Δ*sypQ* mutant (BF237), Δ*litR* Δ*sypQ* mutant (BF245) strains, all of which carried the P*sypA*-*lacZ* reporter, were grown at RT for 22 h without shaking in LC. Cell extracts were assayed for β-galactosidase activity and Miller units were calculated to measure P*sypA* activity. Statistics for panel A were performed using a two-way ANOVA, corrected for multiple comparisons with Šídák's test, ns: not significant, ***: p-value = 0.0007. Statistics for panel B were performed using a one-way ANOVA, corrected for multiple comparisons with Tukey's test, **: p-value < 0.0045, ****: p-value < 0.0001.

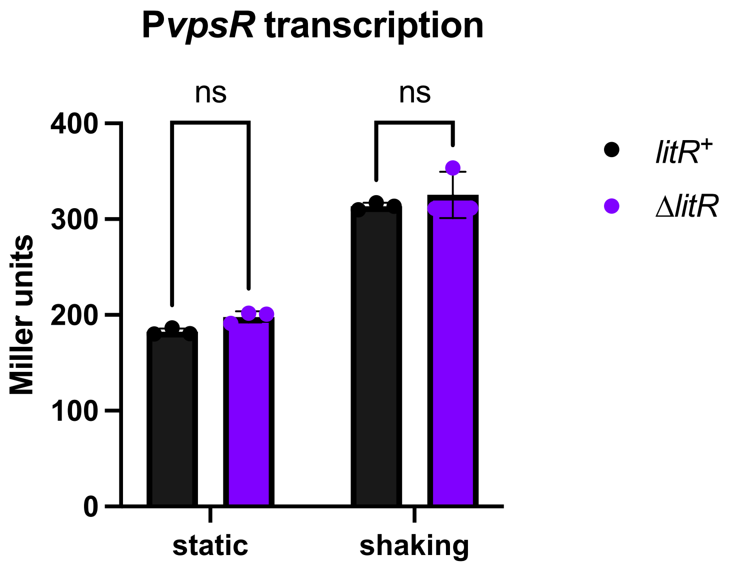

Supplemental Figure 4. LitR does not control *vpsR* transcription. *litR*$^{+}$ (BF315) and Δ*litR* mutant (BF330) strains, both of which carried the Δ*sypQ* mutation and the P*vpsR*-*lacZ* reporter, were incubated with or without shaking at 24°C or RT, respectively, in LC. At 22 h, cell extracts were assessed for β-galactosidase activity and the resulting Miller units were calculated as a measurement of P*vpsR* activity. Statistics were done by two-way ANOVA, uncorrected for multiple comparisons with Fisher's LSD test, ns: not significant.

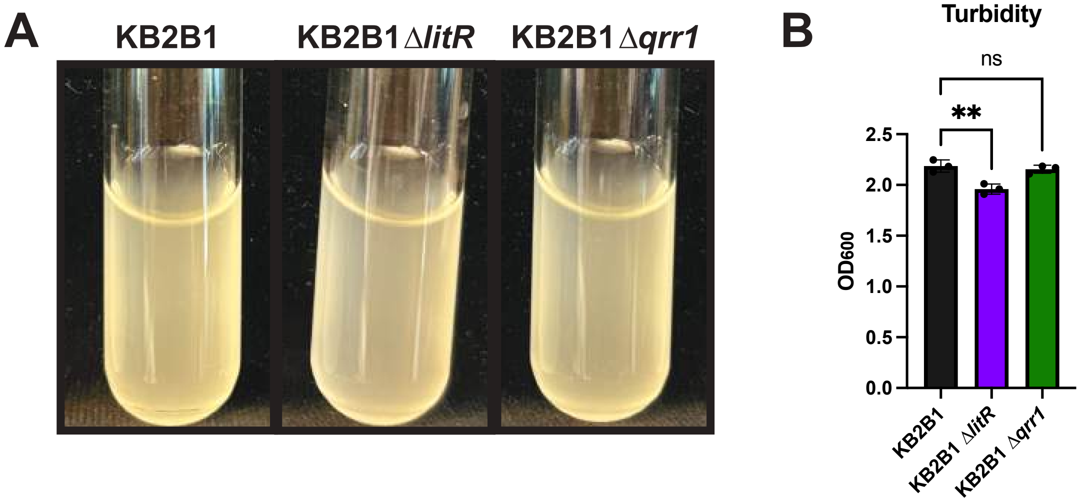

Supplemental Figure 5. LitR does not affect KB2B1 biofilm formation under shaking conditions. (A) WT strain (KB2B1) and its derivatives, Δ*litR* (KV9023), and Δ*qrr1* (KV9105), were imaged after 24 h shaking growth at 24°C in TC. (B) Turbidity of the liquid was measured by OD600 and plotted. Statistics were performed using a one-way ANOVA, corrected for multiple comparisons using Tukey's test, ns: not significant, **: p-value = 0.0038.

Supplemental Table 1. Additional strains used in this study for strain construction

Strains	Genotype[1]	Construction	Reference
**BF162**	IG (Erm^r)::P*sypA-lacZ* Δ*litR*::FRT-Spec^r	TT KV9740 with gKV9806	This study
**BF175**	Δ*sypF*::FRT-Erm^r Δ*litR*::FRT-Spec^r	TT KV9740 with gKV8242	This study
**BF246**	IG (Erm^r)::P*bcsQ-lacZ* Δ*sypQ*::FRT-Cm^r	TT KV9750 with gKV8078	This study
**BF355**	Δ*sypQ*::FRT IG (Erm^r)::P*binK-lacZ*	TT KV9895 with gKV9949	This study
**BF595**	*luxS*::Kan^r Δ*ainS*::FRT-Erm^r	TT CL39 with gKV9367	This study
**KV7371**	IG::P*sypA-lacZ*	N/A	(1)
**KV7952**	Δ*sypE sypF2* Δ*hahK*::FRT-Erm^r	N/A	(2)
**KV8078**	Δ*sypQ*::FRT-Cm^r attTn7::P*bcsQ-lacZ*	N/A	(3)
**KV8232**	IG::Erm^r-trunc Trim^r	N/A	(4)
**KV9367**	Δ*ainS*::FRT-Erm^r	TT ES114 with SOE product amplified with primers 1852 & 2839 (ES114) and 2089 & 2090 (pKV494) and 2840 & 1259 (ES114)	This study
**KV9740**	Δ*litR*::FRT-Spec^r	TT ES114 with SOE product amplified with primers 3017 & 3018 (ES114) and 2089 & 2090 (pKV521) and 3019 & 3020 (ES114)	This study
**KV9750**	IG (Erm^r)::P*bcsQ-lacZ*	TT KV7371 with SOE product amplified with primers 2185 and 2090 (pKV502), 2936 and 2937 (ES114), 2822 and 2876 (KV7371)	This study
**KV9751**	IG (Erm^r)::p*VF_0352-lacZ*	TT KV7371 with SOE product amplified with primers 2185 & 2090 (pKV502) and 2934 & 2935 (ES114) and 2822 & 2876 (KV7371)	This study
**KV9789**	IG::*pVF_0352-lacZ*	Erm^r removed from KV9751	This study
**KV9806**	IG (Erm^r)::P*sypA-lacZ*	TT KV9789 with SOE product amplified with primers 2185 & 2090 (pKV502) and 3083 & 3084 (ES114) and 2822 & 2876 (KV7371)	This study
**KV9838**	IG (Erm^r)::P*nrdR*-P*binK-binK*	TT KV8232 with SOE product amplified with primers 2290 and 2090 (pKV506), 3014 and 2497 (ES114), and 2196 and 1487 (pKV503)	This study
**KV9973**	IG::P*sypA-lacZ* Δ*sypQ*::FRT-Erm^r	TT KV9940 with gKV8191	This study
**KV10050**	IG (Erm^r)::P*litR-litR*	TT KV8232 with SOE product amplified with primers 2185 & 2090 (pKV502) and 3354 & 3310 (ES114) and 2196 & 1487 (pKV503)	This study
**KV10496**	Δ*vpsR*::FRT-Spec^r Δ*litR*::FRT	TT KV10494 with gKV9341	This study

**PMF8**	*litR*::Kan^r	N/A	(5)

[1]Abbreviations: IG, gene inserted at intergenic region between genes *yeiR* and *glmS* along with an FRT scar, with one exception: KV7371 does not contain an FRT scar; IG (Erm), gene inserted between *yeiR* and *glmS* along with FRT-Erm^r; TT, TfoX-mediated transformation using *tfoX*-overexpressing version of indicated strain; trunc, truncation; RBS, idealized ribosome binding site; FLAG, FLAG-epitope tagged; FRT, Flippase Recognition Target; if not followed by an antibiotic resistance gene, then the antibiotic cassette was flipped out leaving an FRT scar within the chromosome; attTn*7*, site used for insertion of genes using transposon Tn*7*.

1. Norsworthy AN, Visick KL. 2015. Signaling between two interacting sensor kinases promotes biofilms and colonization by a bacterial symbiont. Molecular Microbiology 96:233-248.
2. Thompson CM, Marsden AE, Tischler AH, Koo J, Visick KL. 2018. *Vibrio fischeri* biofilm formation prevented by a trio of regulators. Applied and Environmental Microbiology 84:16.
3. Tischler AH, Lie L, Thompson CM, Visick KL. 2018. Discovery of calcium as a biofilm-promoting signal for *Vibrio fischeri* reveals new phenotypes and underlying regulatory complexity. Journal of Bacteriology 200:e00016-18.
4. Visick KL, Hodge-Hanson KM, Tischler AH, Bennett AK, Mastrodomenico V. 2018. Tools for rapid genetic engineering of *Vibrio fischeri*. Applied and Environmental Microbiology 84:AEM.00850-18.
5. Fidopiastis PM, Miyamoto CM, Jobling MG, Meighen EA, Ruby EG. 2002. LitR, a new transcriptional activator in *Vibrio fischeri*, regulates luminescence and symbiotic light organ colonization. Molecular Microbiology 45:131-143.
